# CD71^+^VISTA^+^ erythroid cells promote the development and function of regulatory T cells through TGF-β

**DOI:** 10.1371/journal.pbio.2006649

**Published:** 2018-12-14

**Authors:** Shima Shahbaz, Najmeh Bozorgmehr, Petya Koleva, Afshin Namdar, Juan Jovel, Roy A. Fava, Shokrollah Elahi

**Affiliations:** 1 Department of Dentistry, Faculty of Medicine and Dentistry, University of Alberta, Edmonton, Canada; 2 The Applied Genomics Core, Office of Research, University of Alberta, Edmonton, Canada; 3 Department of Veterans Affairs Medical Center, Research Service, White River Junction, Vermont, United States of America; 4 Department of Medicine, Geisel School of Medicine at Dartmouth, Dartmouth Hitchcock Medical Center, Lebanon, New Hampshire, United States of America; 5 Department of Medical Microbiology and Immunology, Faculty of Medicine and Dentistry, University of Alberta, Edmonton, Canada; National Jewish Health, United States of America

## Abstract

Cell-surface transferrin receptor (CD71^+^) erythroid cells are abundant in newborns with immunomodulatory properties. Here, we show that neonatal CD71^+^ erythroid cells express significant levels of V-domain Immunoglobulin (Ig) Suppressor of T Cell Activation (VISTA) and, via constitutive production of transforming growth factor (TGF)- β, play a pivotal role in promotion of naïve CD4^+^ T cells into regulatory T cells (Tregs). Interestingly, we discovered that CD71^+^VISTA^+^ erythroid cells produce significantly higher levels of TGF-β compared to CD71^+^VISTA^−^ erythroid cells and CD71^+^ erythroid cells from the VISTA knock-out (KO) mice. As a result, CD71^+^VISTA^+^ erythroid cells—compared to CD71^+^VISTA^−^ and CD71^+^ erythroid cells from the VISTA KO mice—significantly exceed promotion of naïve CD4^+^ T cells into induced Tregs (iTreg) via TGF-β in vitro. However, depletion of CD71^+^ erythroid cells had no significant effects on the frequency of Tregs in vivo. Surprisingly, we observed that the remaining and/or newly generated CD71^+^ erythroid cells following anti-CD71 antibody administration exhibit a different gene expression profile, evidenced by the up-regulation of VISTA, TGF-β1, TGF-β2, and program death ligand-1 (PDL-1), which may account as a compensatory mechanism for the maintenance of Treg population. We also observed that iTreg development by CD71^+^ erythroid cells is mediated through the inhibition of key signaling molecules phosphorylated protein kinase B (phospho-Akt) and phosphorylated mechanistic target of rapamycin (phospho-mTOR). Finally, we found that elimination of Tregs using forkhead box P3 (FOXP3)-diptheria toxin receptor (DTR) mice resulted in a significant expansion in the frequency of CD71^+^ erythroid cells in vivo. Collectively, these studies provide a novel, to our knowledge, insight into the cross-talk between CD71^+^ erythroid cells and Tregs in newborns. Our results highlight the biological role of CD71^+^ erythroid cells in the neonatal period and possibly beyond.

## Author summary

The primary role of the red blood cells is to transport oxygen, but we know relatively little about the other functions they perform. Following maturation, red blood cells exit the bone marrow and enter blood circulation. Their immature counterparts are normally absent or in very low frequency in the blood of healthy adults. However, we showed previously that immature red blood cells are abundant in the spleens of neonatal mice and in human umbilical cord blood and that these cells possess immunological properties. In this report, we studied a subset of neonatal immature red blood cells that express a protein called V-domain Immunoglobulin (Ig) Suppressor of T Cell Activation (VISTA) on their surface. We found that the presence of VISTA enables the cells to repeatedly produce the regulatory cytokine TGF-β. TGF-β induces a subset of naïve lymphocytes—the CD4^+^ T cells—and converts them into regulatory T cells, also known as Tregs. Tregs modulate and suppress other immune cells. Our studies provide novel insights, to our knowledge, into the immunological role of immature red blood cells in newborns.

## Introduction

Reticulocytes originate in bone marrow (BM) from erythroblasts by the process of nuclear extrusion and are released into the blood, where they further mature into erythrocytes (red blood cells). Reticulocytes contain RNA, mitochondria, endoplasmic reticulum, and ribosomes. As reticulocyte maturation progresses, the nucleus vanishes, protein synthesis ceases, and cell-surface transferrin receptor (CD71) disappears [1].

The main function of vertebrate erythrocytes has been considered to be oxygen transporters; however, other functions such as interactions with immune cells and immunomodulatory properties have also been attributed to their immature counterparts [2,3]. In 1979, for the first time, Pavia and colleagues demonstrated the immunosuppressive effects of murine neonatal splenocytes on adult cells [4,5]. Subsequent studies towards understanding the immunomodulatory mechanism of these cells showed that TGF-β [6] and direct cell–cell interactions might partially be involved for their immunosuppressive effects [7]. Moreover, it has been suggested that erythroid precursors promote polarization of naive CD4^+^ T cells to different T cell subtypes [3,8] and may alter the T-helper 1/2 (Th1/Th2) cytokine balance toward a Th2 phenotype in newborns [9]. In agreement, we have recently discovered that CD71^+^ erythroid cells are enriched in newborns and, because of their immunosuppressive properties, impair both the innate and adaptive immune responses in newborns [10–12]. These cells coexpress CD71 (transferrin receptor) and glycophorin-A–associated protein (TER119; the erythroid lineage marker) in mice or CD71 and CD235a (the erythroid lineage marker) in humans [11]. In addition, we have shown that CD71^+^ erythroid cells express arginase-2, and this enzymatic activity is required for their immunosuppressive activity [10,11]. Overall, our previous studies have revealed that neonatal vulnerability to infection results from the temporary existence of immunosuppressive CD71^+^ erythroid cells. In agreement, a recent study has shown a marked expansion of erythroid precursors (CD71^+^ TER119^+^) in murine spleens following *Salmonella* infection and demonstrated that presence of these cells was associated with enhanced bacterial persistence [9]. More recently, the immunomodulatory properties of CD71^+^ erythroid cells in cord blood of preterm versus full-term newborns have been investigated [13]. Although we have shown that CD71^+^ erythroid cells express arginase-2, it is unclear whether these erythroid cells express surface molecules such as inhibitory receptors in order to execute their immunosuppressive/immunoregulatory properties.

Inhibitory receptors or immune checkpoints regulate T cell function by restraining initial T cell activation that otherwise can be potentially pathogenic for the host. However, sustained expression of coinhibitory receptors such as program death-1 (PD-1), cytotoxic T-lymphocyte-associated protein 4 (CTLA-4), T-cell immunoglobulin and mucin-domain containing-3 (TIM-3), and lymphocyte activation 3 (LAG-3) are hallmarks of dysfunctional T cells [14–16]. Coinhibitory receptors, upon interaction with their ligands on antigen-presenting cells (APCs) and/or tumor cells, mediate immune suppression [14,15]. V-domain Ig Suppressor of T Cell Activation (VISTA), a newly discovered inhibitory receptor, is a transmembrane immunoglobulin (Ig) super family also known as Programmed Death-1 Homologue-1 (*PD-1H*), *vsir*, *Gi24*, *Dies1*, *DD1α*, *c10orf54*, and *SISP1* [17–19]. VISTA messenger RNA (mRNA) is expressed predominantly in hematopoietic and less in nonhematopoietic tissues. Within the hematopoietic compartment, VISTA is expressed on dendritic cells, monocytes, neutrophils, and natural killer (NK) cells, as well as naïve T cells and regulatory T cells (Tregs), but not on B cells [17–19]. So far, several studies have shown that VISTA has immunomodulatory functions, regardless of its regulatory role in differentiation of osteoblast, adipocyte, and embryonic stem cells [20–23] and cell apoptosis [24].

VISTA is unique among other immunoglobulin superfamily molecules but lacks classic immunoreceptor tyrosine-based inhibition motifs (ITIMs) or immunoreceptor tyrosine-based switch motifs (ITSMs) [18]. This explains the dual role for VISTA on APCs or T cells as a ligand or receptor, respectively. The inhibitory function of VISTA has widely been studied and supported by several in vitro and in vivo reports [18,19,25,26]. These studies have revealed that agonistic monoclonal antibodies to VISTA induce inhibitory response [26], in contrast to antagonistic antibodies that promote stimulatory action [18,25,27]. Although VISTA is considered as an inhibitory molecule, no evidence of overt autoimmunity was reported in VISTA knock-out (KO) mice. However, T-cell–activation phenotype and accelerated aging is reported in these VISTA KO mice [26,28,29]. Furthermore, the anti-inflammatory role of VISTA has been proved in different studies using mouse models of Graft Versus Host Disease (GVHD), Concanavalin A (Con-A)–induced hepatitis [28,29], and experimental autoimmune encephalomyelitis [27]. Moreover, it has been shown that VISTA has an anti-inflammatory role via regulation of the interleukin-23/17 (IL-23/IL-17) axis [30], and VISTA blockade in various animal tumor models has shown promising results in terms of enhanced T cell response against the tumor [25,26,31,32]. However, the mechanism(s) underlying the role of VISTA in stem cell differentiation and/or immunomodulation have yet to be well elucidated.

Some reports indicated that VISTA induces stem cell differentiation via interaction with TGF-β family members including Bone Morphogenic Protein-4 (BMP4). In the TGF-β downstream pathway, VISTA promotes BMP4 signaling through activation of SMAD 1, 5, and 8; VISTA forms a complex with SMAD 4 and regulates target gene expression toward stem cell differentiation [20,22,33]. Moreover, VISTA with microRNA-125a (miR-125a) generates a regulatory loop that modulates BMP4 signaling on stem cell differentiation in vitro [22]. Other studies suggest that VISTA is constitutively expressed on Tregs and plays an important role in their function [20,22]. As such, VISTA improves the induction of Foxp3^+^-induced Tregs (iTregs) in the presence of TGF-β in both mice and human CD4^+^ T cells in vitro [18,32]. A recent study using wild-type (WT) and VISTA KO mice has shown that VISTA is required for de novo induction and expansion of iTregs from naïve T cells [34]. Since some reports indicated a role for VISTA in stem cell differentiation, we aimed to investigate the expression and function of VISTA in CD71^+^ erythroid cells.

In this study, for the very first time to our knowledge, we have shown that CD71^+^ erythroid cells express significant levels of VISTA, and more importantly, the CD71^+^VISTA^+^ subpopulation, compared to the CD71^+^VISTA^−^ subpopulation, produces significant levels of TGF-β. In addition, we have shown defects in CD71^+^ erythroid cells from VISTA KO mice in terms of TGF-β production ability. We also demonstrate that CD71^+^VISTA^+^ erythroid cells via TGF-β enhance iTreg induction in vitro. Thus, our studies provide a novel insight, to our knowledge, into the role of CD71^+^ erythroid cells in immune regulation and cross-talk with Tregs.

## Results

### Transcriptional profile of CD71^+^ erythroid cells

**Fig 1:**
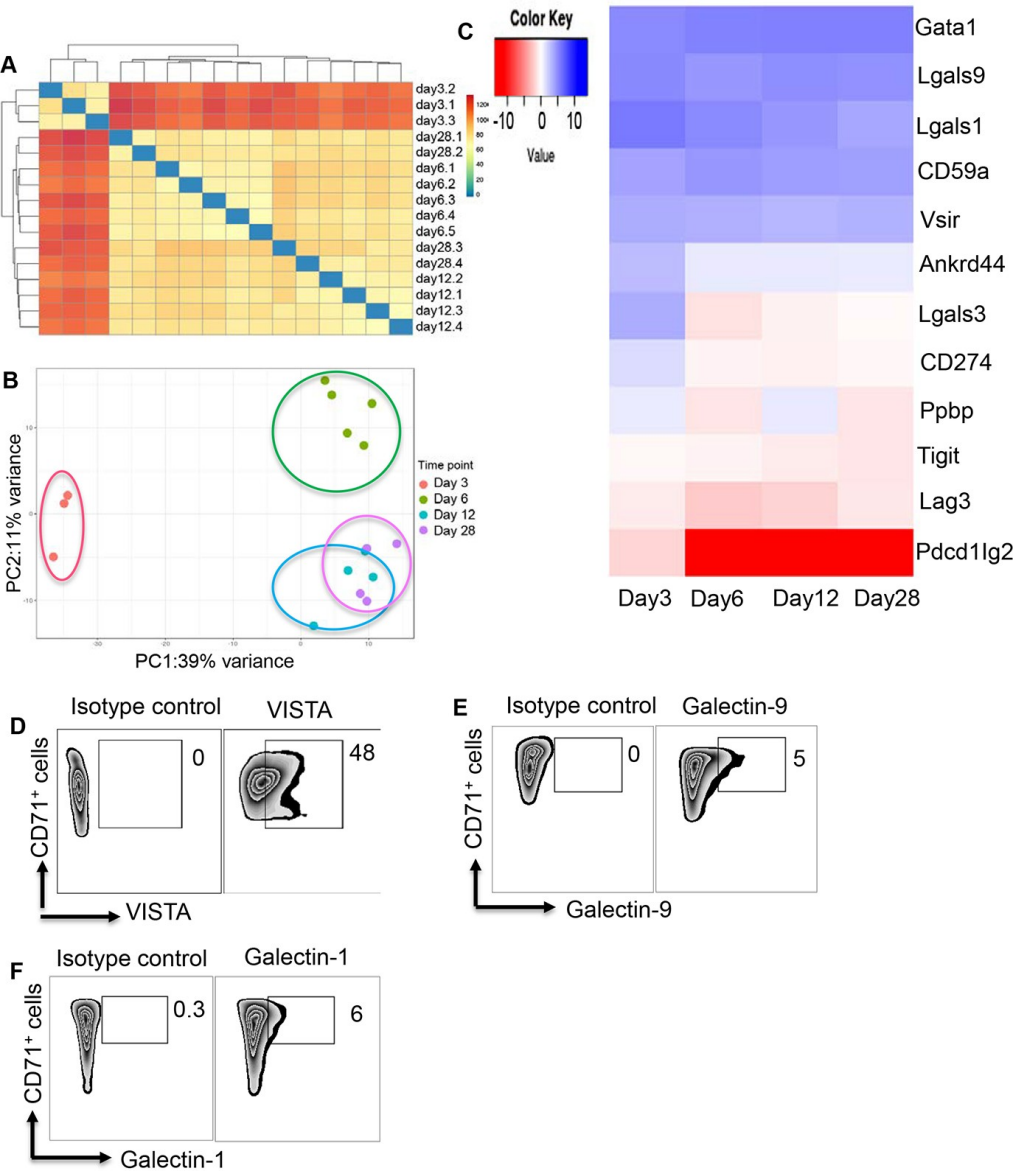
Gene expression characteristic of CD71^+^ erythroid cells. (A) Hierarchical clustering on Euclidian distances showing different gene expression profile in CD71^+^ erythroid cells. (B) PCA on the Euclidian distances between CD71^+^ erythroid cells in different ages. (C) Expression of genes associated with inhibitory receptors/ligands is shown using heatmap. (D) Representative plots showing expression of VISTA, (E) Lgals9, and (F) Lgals1 among CD71^+^ erythroid cells compared to corresponding isotype controls. The underlying data can be found in S1 Data. Ankrd44, ankyrin repeat domain 44 gene; CD59a, glycoprotein-A–associated gene; CD71, cell-surface transferrin receptor; CD274, Program death ligand-1; Gata1, erythroid transcription factor; Ig, immunoglobulin; ITIM, immunoreceptor tyrosine-based inhibition motif; Lag3, lymphocyte activation 3; Lgals1, galectin-1 gene; Lgals3, galectin-3 gene; Lgals9, galectin-9 gene; PC, principal component; PCA, principal component analysis; Pdcd1Ig2, Programmed Cell Death 1 Ligand 2 gene; Ppbp, pro-platelet basic protein gene; Tigit, T cell immunoreceptor with Ig and ITIM domains; VISTA, V-domain Ig Suppressor of T Cell Activation; Vsir, VISTA gene.

In order to determine the transcriptional profile of CD71^+^ erythroid cells over time, we conducted RNA sequencing (RNAseq) analysis on total RNA extracted from enriched CD71^+^ erythroid cells from the spleen of 3-, 6-, 12-, and 28-day-old BALB/c mice. When hierarchical clustering was conducted on Euclidian distances between samples, day 3 CD71^+^ erythroid cells clearly showed a different gene expression profile than the rest of the time points (Fig 1A). For the most part, day 6 and day 12 samples formed separated branches on a dendrogram, while day 28 samples exhibited a more erratic distribution along such dendrograms (Fig 1A). Those results are partially recapitulated in principal component analysis (PCA) on the Euclidian distances between samples. In essence, day 3 CD71^+^ erythroid cells clearly separated from the rest of the samples along the first principal component, and day 6 samples formed a rather discrete cluster. Day 12 and day 28 samples intermingled (Fig 1B). In summary, the transcriptional profile of day 3 samples is clearly distinct from the rest of the samples. Day 6 CD71^+^ erythroid cells were found to have a transcriptional profile somewhat different from day 12 and day 28 samples, and the latter two groups showed a similar transcriptional profile. This suggests that a rather drastic change in the gene expression program of CD71^+^ erythroid cells takes place early during mice development, and no other major changes are observed at later time points.

We have previously shown that CD71^+^ erythroid cells are enriched in C57BL/6 newborn mice and gradually disappear by 3 weeks of age [11]. To determine the frequency of CD71^+^ erythroid cells in the spleens of three different mice strains (BALB/c, C57BL/6, and filial 1 hybrid [F1]), we compared their presence at different ages (S1A Fig). The percentages of CD71^+^ erythroid cells in BALB/c peaked between days 6 and 9, followed by a gradual decline at later time points to reach levels comparable to adult mice by day 28 (S1A and S1B Fig). A similar pattern was observed for the frequency of CD71^+^ erythroid cells in C57BL/6 and F1 mice, respectively (S1C and S1D Fig). Of note, although the frequency of CD71^+^ erythroid cells reached adult levels by day 21 in Cincinnati [11], these cells remained for a week longer in the neonatal mice in Edmonton. This might be due to differences in microbiome or other physiological and environmental factors.

We decided to determine gene expression of different inhibitory molecules and/or their ligands in CD71^+^ erythroid cells. Our RNAseq data indicated high gene expression for galectin-9 (Lgals9), galectin-1 (Lgals1), and VISTA (vsir) in CD71^+^ erythroid cells, respectively (Fig 1C). However, other inhibitory receptors were absent or expressed at very low levels (e.g., galectin-3 [Lgals3], TIGIT, LAG-3, and PD-L1) (Fig 1C). To confirm our RNAseq data, we measured surface expression of highly expressed inhibitory molecules (Lgals9, Lgals1, and VISTA) on CD71^+^ erythroid cells. We found that VISTA was highly expressed on CD71^+^ erythroid cells compared to Lgals9 and Lgals1 (Fig 1D–1F).

### Identification of preferential VISTA expression on CD71^+^ erythroid cells

**Fig 2:**
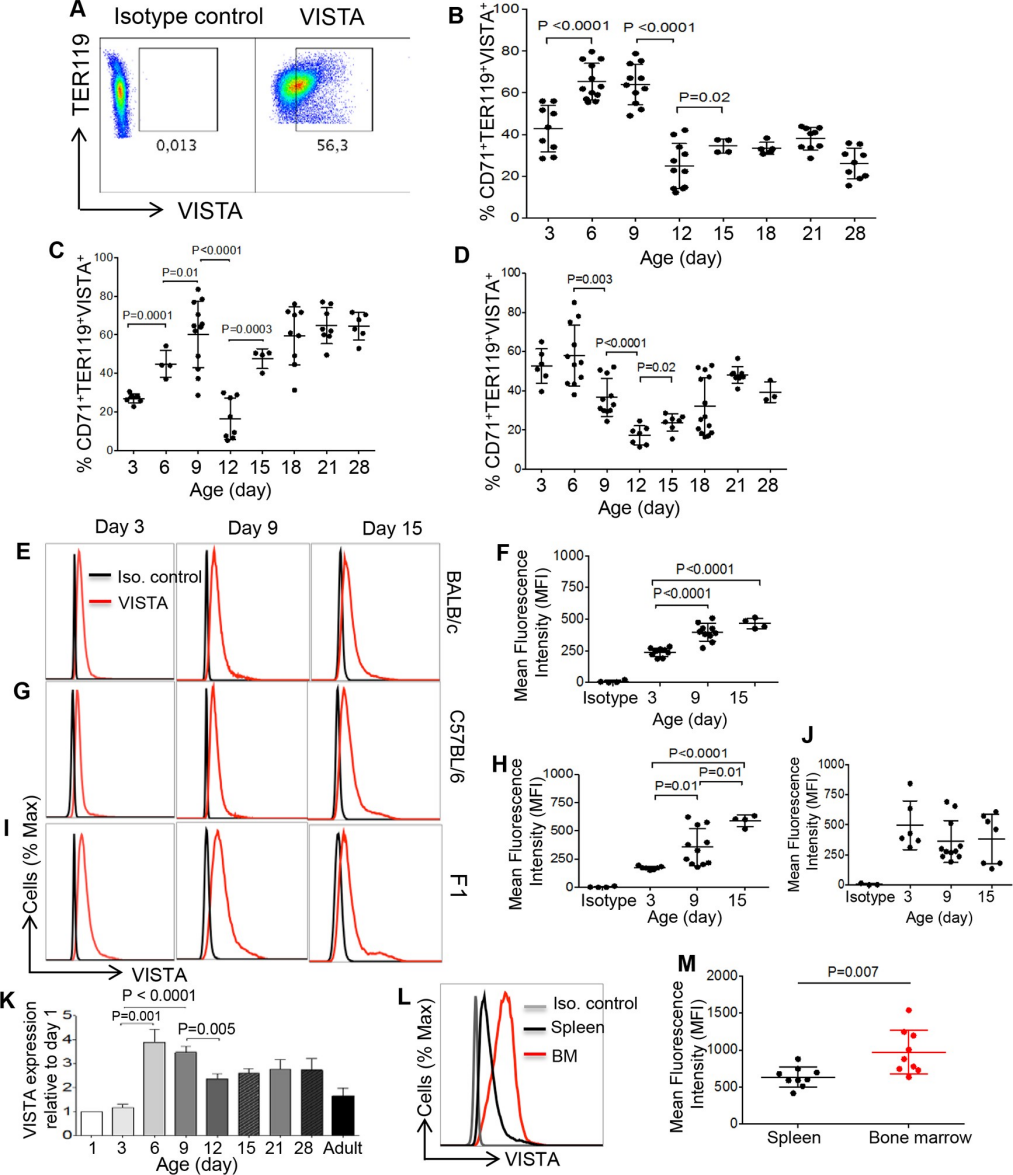
CD71^+^ erythroid cells express VISTA. (A) Representative dot plots showing VISTA expression by CD71^+^ erythroid cells from a day 3 newborn mouse. (B) Percentages of CD71^+^VISTA^+^ erythroid cells among splenocytes of BALB/c, (C) C57BL/6, and (D) F1 mice are shown at different ages, respectively. (E) Representative histogram showing VISTA expression (red line) or isotype control (black line) and (F) the MFI of VISTA among CD71^+^ erythroid cells in BALB/c mice. (G) Representative histogram showing VISTA expression (red line) or isotype control (black line) and (H) the MFI of VISTA among CD71^+^ erythroid cells in B57BL/6 mice. (I) Representative histogram showing VISTA expression (red line) or isotype control (black line) and (J) the MFI of VISTA among CD71^+^ erythroid cells in F1 mice. (K) RT-PCR of VISTA gene expression among neonatal CD71^+^ erythroid cells in BALB/c mice at different ages. (L) Representative histogram of VISTA expression among neonatal CD71^+^ erythroid cells originated from either spleen or BM. (M) The MFI for VISTA among neonatal CD71^+^ erythroid cells from spleen versus BM of neonatal mice. Each point represents data from an individual mouse, representative of at least 2–3 independent experiments. Bar, mean ± one standard error. The underlying data can be found in S1 Data. BM, bone marrow; CD71, cell-surface transferrin receptor; F1, filial 1 hybrid mice; Ig, immunoglobulin; Iso., isotype; MFI, mean fluorescence intensity; RT-PCR, reverse transcription PCR; TER119, glycophorin-A–associated protein; VISTA, V-domain Ig Suppressor of T Cell Activation.

Therefore, we aimed to investigate the role of VISTA in CD71^+^ erythroid cell function. As shown in Fig 2A and 2B, approximately 40% of CD71^+^ erythroid cells expressed VISTA in neonatal BALB/c mice at day 3. VISTA expression levels peaked on days 6–9 (>60%), followed by a significant decline at day 12, which remained at similar levels thereafter. In contrast, in the C57BL/6 mouse strain, there was a lower expression level for VISTA on CD71^+^ erythroid cells in 3-day-old mice (<30% compared to >40% in BALB/c mice) and significantly increased in 6-day-old mice, still lower than the same age in BALB/c mice (approximately 45% compared to approximately 65%) but peaked to a similar level as BALB/c mice at day 9, followed by a significant decline at day 12 (Fig 2C). Contrary to BALB/c mice, VISTA expression was significantly increased at days 15–18 in C57BL/6 mice and then remained constant until day 28 (Fig 2C). In F1 mice, approximately 50% of CD71^+^ erythroid cells expressed VISTA at day 3, and the expression level of VISTA peaked at day 6 but, interestingly, was reduced at day 9 compared to BALB/c and C57BL/6 mice (Fig 2D). The expression level of VISTA then decreased abruptly to reach the minimum level at day 12, after which there was a significant increase at day 15, which remained at a similar level until day 28 (Fig 2D).

In addition, the intensity of VISTA expression on CD71^+^ erythroid cells was measured at different time points. We observed that the mean fluorescence intensity (MFI) of VISTA was significantly increased on CD71^+^ erythroid cells of neonatal mice at days 9 and 15 compared to day 3 in both BALB/c (Fig 2E and 2F) and B57BL/6 mice (Fig 2G and 2H). However, the intensity of VISTA on CD71^+^ erythroid cells in F1 mice remained unchanged at days 3, 9, and 15 (Fig 2I and 2J). In order to confirm the RNAseq data, we evaluated VISTA gene expression levels in mice at different ages. Consistent with the protein levels, VISTA mRNA expression levels were significantly higher at days 6 and 9 compared to day 3, followed by a significant drop at day 12 in BALB/c mice (Fig 2K).

We further analyzed the expression of VISTA among CD71^+^ erythroid cells obtained from the BM compared to the spleen. Interestingly, we found that the MFI of VISTA on CD71^+^ erythroid cells from BM was significantly higher than their counterparts in the spleen (Fig 2L and 2M). Similar results were obtained for the percentages of VISTA^+^CD71^+^ erythroid cells in the BM versus the spleen (S1E Fig).

Although adult mice have very low percentages of CD71^+^ erythroid cells in their spleens compared to neonates (S1F Fig), these cells expressed high levels of VISTA, but no difference in its expression level was observed when male and female mice were compared (S1G Fig).

### CD71^+^VISTA^+^ erythroid cells produce TGF-β

**Fig 3:**
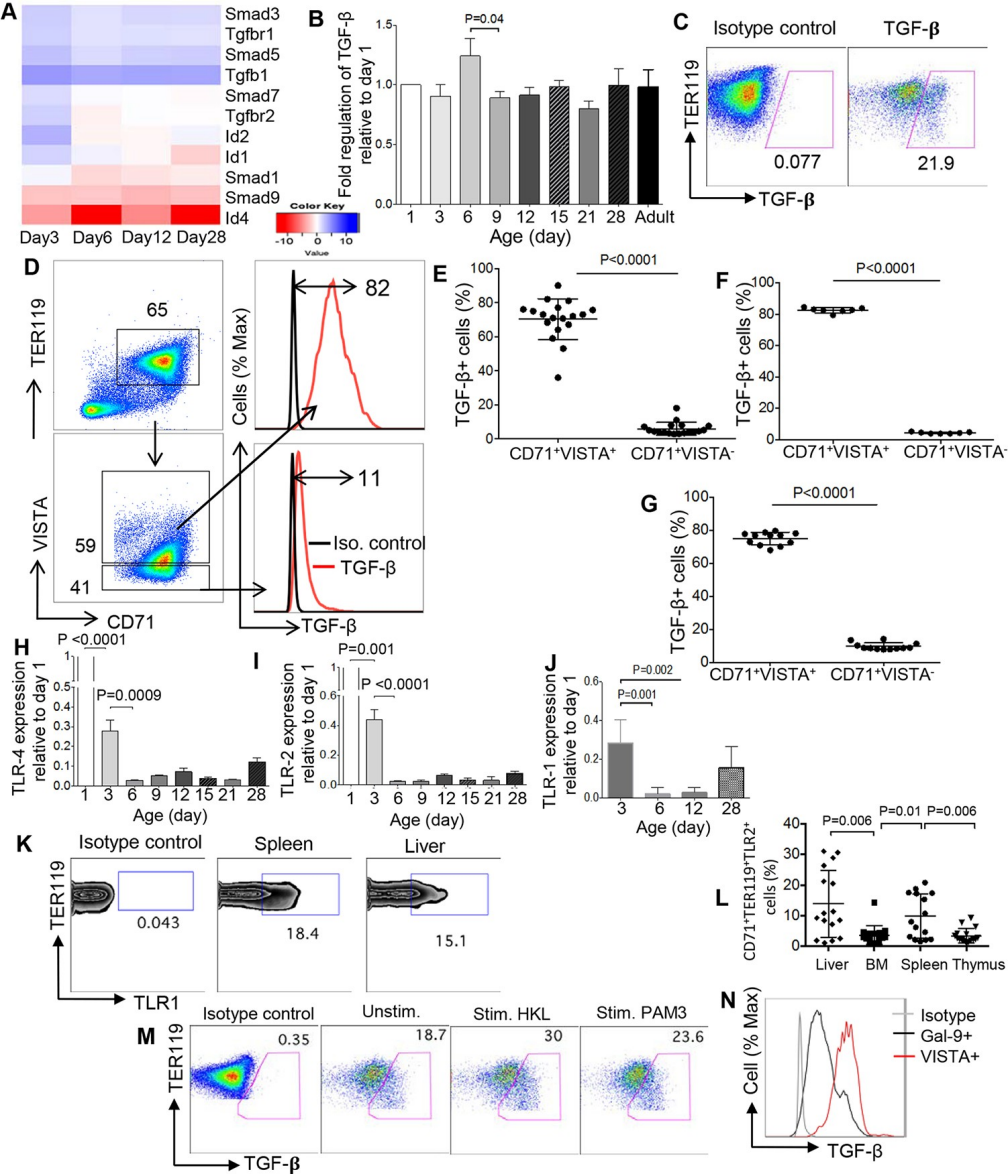
CD71^+^VISTA^+^ cells produce more TGF-β compared to CD71^+^VISTA^−^ cells. (A) Expression of genes associated with TGF-β is shown using heatmap. (B) TGF-β gene expression in isolated CD71^+^ erythroid cells from BALB/c mice at different ages using qPCR. (C) Representative flow cytometry dot plot showing TGF-β secretion by CD71^+^ erythroid cells. (D) Gating strategy and the representative dot plots/histogram showing TGF-β^+^ cells among CD71^+^VISTA^+^ cells compared to CD71^+^VISTA^−^ cells in 6-day-old BALB/c mouse. (E) Percentages of TGF-β^+^ cells among CD71^+^VISTA^+^ cells compared to CD71^+^VISTA^−^ cells in 6-day-old neonatal BALB/c mice or (F) C57BL/6 mice and (G) F1 mice. (H) Expression of TLR4, (I) TLR2, and (J) TLR1 genes among CD71^+^ erythroid cells from 3-day-old mice. (K) Representative flow cytometry zebra plots showing expression of TLR1 among spleen and liver CD71^+^ erythroid cells in a 3-day-old neonatal mouse. (L) Percentages of TLR1 surface expression among CD71^+^ erythroid cells from the liver, BM, spleen, and thymus of 3-day-old BALB/c mice. (M) Representative dot plots showing TGF-β^+^ cells among neonatal CD71^+^ erythroid cells before and after stimulation with Pam_3_CSK_4_ or HL Lm. (N) Representative flow cytometry histogram showing TGF-β expression by Lgals9^+^ versus VISTA^+^ CD71^+^ erythroid cells. Data are representative of at least three independent experiments. Bar, mean ± one standard error. The underlying data can be found in S1 Data. BM, bone marrow; CD71, cell-surface transferrin receptor; HK, heat killed; Ig, immunoglobulin; Lm, *Listeria monocytogenes*; Pam_3_CSK_4_, N-palmitoyl-S-dipalmitoylglyceryl Cys-Ser-(Lys) 4; qPCR, quantitative PCR; Stim., stimulated; TER119, glycophorin-A–associated protein; TGF-β, transforming growth factor beta; TLR, Toll-like receptor; Unstim., unstimulated; VISTA, V-domain Ig Suppressor of T Cell Activation.

What is the role of VISTA on CD71^+^ erythroid cells? Given the association of VISTA with TGF-β family members, including BMP4, we investigated TGF-β–associated gene expression profile in CD71^+^ erythroid cells. Interestingly, we found TGF-β1, Smad3, Smad5, and TGF-β receptor 1 (r1) genes were markedly increased (Fig 3A). Increased TGF-β expression by CD71^+^ erythroid cells was also confirmed by quantitative polymerase chain reaction (qPCR) and flow cytometry (Fig 3B and 3C).

We then investigated whether there was any association between VISTA expression and TGF-β production. We found that CD71^+^VISTA^+^ cells constitutively produce significantly higher levels of TGF-β compared to CD71^+^VISTA^−^ cells at different ages in BALB/c mice (Fig 3D and 3E). Similar results were observed among neonatal CD71^+^ erythroid cells in C57BL/6 and F1 mice (Fig 3F and 3G, respectively). To determine whether we can use Toll-like receptor agonists to enhance TGF-β secretion from CD71^+^ erythroid cells, we analyzed the expression of different toll-like receptors on these cells. We found that CD71^+^ erythroid cells express Toll-like receptor (TLR) 1, TLR2, and TLR-4 genes. However, their expression levels dropped significantly at day 3 compared to day 1, and by day 6, their expression levels became almost undetectable and remained at very low levels thereafter (Fig 3H, 3I and 3J). Therefore, we decided to measure the expression of TLRs among neonatal CD71^+^ erythroid cells at day 3 by flow cytometry. As shown in Fig 3K and 3L, we observed significantly higher surface expression of TLR1 among neonatal CD71^+^ erythroid cells in liver and spleen compared to their counterparts in the BM and thymus. Since CD71^+^ erythroid cells express TLR1, we then stimulated CD71^+^ erythroid cells with N-palmitoyl-S-dipalmitoylglyceryl (Pam_3_) Cys-Ser- (Lys) 4 (CSK_4_), a TLR-1 agonist, and heat-killed (HK) *Listeria monocytogenes* (Lm), a TLR-2 agonist. As is shown in Fig 3M, stimulation by HK Lm and/or Pam_3_CSK_4_ enhanced production of TGF-β among CD71^+^ erythroid cells. Of note, since we have shown a small portion of CD71^+^ erythroid cells express Lgals1 and Lgals9 on their surface (Fig 1E and 1F), we compared TGF-β production by Lgals9^+^ versus Lgals9^−^ cells. We found that Lgals9^+^ cells produce higher levels of TGF-β compared to Lgals9^−^ CD71^+^ erythroid cells. However, VISTA^+^CD71^+^ erythroid cells surpass Lgals9^+^ cells in terms of TGF-β production (Fig 3N). In addition, we observed coexpression of VISTA with Lgals1 and Lgals9 on a small portion of CD71^+^ erythroid cells (S1H and S1I Fig). Furthermore, we investigated the expression of glycoprotein A repetitions predominant (GARP) on VISTA^+^ versus VISTA^−^ CD71^+^ erythroid cells. Although VISTA^+^ cells appear to express slightly higher percentages of GARP, it did not reach significance (S1J and S1K Fig).

### CD71^+^VISTA^+^ erythroid cells promote and sustain FOXP3 expression and the suppressive function of iTregs in vitro

**Fig 4:**
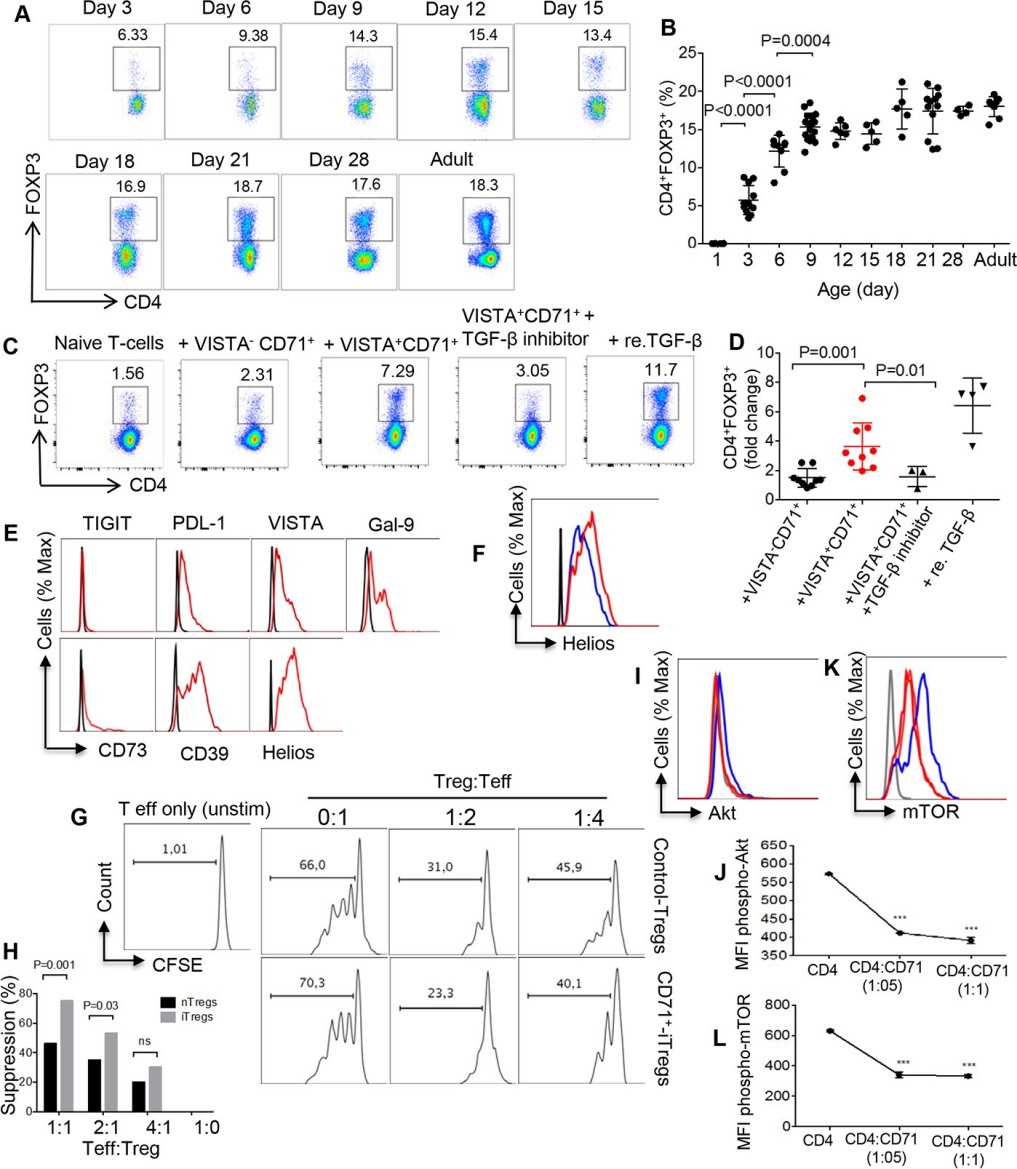
CD71^+^VISTA^+^ cells enhance iTreg development in newborn mice. (A) Representative flow cytometry dot plots showing CD4^+^FOXP3^+^ Tregs among neonatal splenocytes. (B) Percentages of Tregs among neonatal splenocytes in BALB/c mice at different ages. (C) Representative flow cytometry dot plots showing percentages of iTregs in the presence of either CD71^+^VISTA^+^ or CD71^+^VISTA^−^ erythroid cells, TGF-β blocker, and re. TGF-β. (D) Cumulative data showing fold change in CD4^+^FOXP^+^ Tregs in the presence of either CD71^+^VISTA^+^ or CD71^+^VISTA^−^ erythroid cells. (E) The expression of TIGIT, PDL-1, VISTA, Lgals9, CD73, CD39, and Helios (red lines) on the iTreg were determined by flow cytometry (isotype controls, black line). (F) The expression of Helios on the iTregs (red line) was compared to the spleen Tregs (blue line). (G and H) Assessing the suppressive function of iTregs in vitro. Naïve CD4^+^ T cells were cocultured with CD71^+^VISTA^+^ erythroid cells to promote iTregs following activation with anti-CD3/CD28 antibodies. The iTregs were enriched along total spleen Tregs and cocultured with Teff as shown in the ratios. (I and J) Phospho-Akt (K and L) and phospho-mTOR at 18 h after coculture of CD71^+^ erythroid cells with naïve CD4^+^ T cells, following stimulation with anti-CD3/CD28 antibodies (gray line isotype control, red line CD4:CD71, and blue line naïve CD4^+^ T cells). Asterisks are used to indicate significance (*p* < 0.0001). Data are representative of at least two to three independent experiments. Bar, mean ± one standard error. The underlying data can be found in S1 Data. BALB/c, mouse strain; CD71, cell-surface transferrin receptor; CFSE, Carboxyfluorescein succinimidyl ester; FOXP3, forkhead box P3; Ig, immunoglobulin; iTreg, induced Treg; Lgals9, galectin-9; MFI, mean fluorescence intensity; phospho-Akt, phosphorylated-Akt; PDL-1, program death ligand-1; phospho-mTOR, phosphorylated-mTOR; re. TGF-β, recombinant TGF-β; Teff, effector T cell; TGF-β, transforming growth factor beta; TIGIT, T cell immunoreceptor with Ig and ITIM domains; Treg, regulatory T cell; VISTA, V-domain Ig Suppressor of T Cell Activation.

We first determined the frequency of Tregs at different ages in newborn BALB/c mice. Tregs can be detected in the spleen of the newborn mouse starting at day 3. The percentage of Tregs rose significantly thereafter until day 9, after which there was a gradual increase to reach the adult levels (Fig 4A and 4B). However, the absolute number of Tregs expanded gradually from the day 3 to day 18 and then significantly increased at days 21 and 28 before reaching the adult level (S1L Fig). As shown in Fig 4B, we observed a dramatic increase in the percentages of Tregs at days 6 and 9, which coincided with the maximal abundance of CD71^+^ erythroid cells in newborn mice (S1A–S1D Fig). Based on these observations, we decided to determine whether there was cross-talk between these two immunosuppressive cell populations in the neonatal period. As we have shown in Fig 3D, VISTA^+^CD71^+^ compared to VISTA^−^CD71^+^ erythroid cells produce significantly higher levels of TGF-β. Therefore, we isolated VISTA^+^CD71^+^ and VISTA^−^CD71^+^ erythroid cells and cocultured each subpopulation with the isolated naïve CD4^+^ T cells from adult mice at a 1:1 ratio. After 4 days, we found that VISTA^+^CD71^+^ erythroid cells promoted naïve CD4^+^ T cell conversion into iTregs (Fig 4C and 4D). Our data demonstrate that VISTA^+^CD71^+^ erythroid cells significantly expanded Tregs mainly via TGF-β production because blocking TGF-β significantly abrogated the effects of VISTA^+^CD71^+^ on iTreg induction (Fig 4C and 4D). In contrast, the effects of VISTA^−^CD71^+^ subpopulation on the induction of Tregs was significantly lower (Fig 4C and 4D), possibly due to the lower TGF-β production by these cells. Next, the phenotype of iTregs was analyzed, and we found iTregs do not express TIGIT and express low CD73 but high PDL-1, VISTA, Lgals9, and CD39 (Fig 4E). Interestingly, we found both natural and iTregs express helios (Fig 4F), which agrees with the recent finding claiming that helios should not be considered as a marker of natural Tregs [35,36]. Furthermore, we found that these iTregs significantly inhibited the proliferative capacity of effector T cells in a dose-dependent manner when compared to the spleen Tregs (Fig 4G and 4H). Other studies have shown that antagonizing the Akt signaling pathway is required for Treg induction [37,38]. To test this, we cultured naïve T cells in the presence or absence of CD71^+^ erythroid cells for 18 h and then measured phosphorylation of Akt and mTOR. Intracellular staining for phospho-Akt and phospho-mTOR revealed significant reduction in Akt (Fig 4I and 4J) and mTOR phosphorylation (Fig 4K and 4L) levels when naïve T cells were cocultured with CD71^+^ erythroid cells.

In addition, we decided to investigate whether CD71^+^ erythroid cells impact FOXP3 induction via arginase-2 expression in naïve CD4^+^ T cells in vitro. We have reported elsewhere that CD71^+^ erythroid cells utilize this enzyme as one of their immunosuppressive mechanisms [11]. Therefore, we measured FOXP3 expression on naïve CD4^+^ T cells in the presence of CD71^+^ erythroid cells and L-arginine. Our observations showed that neutralization of arginase-2 by L-arginine supplementation did not impact Treg frequency (S1M Fig).

### CD71^+^ erythroid cells from VISTA KO mice produce lower levels of TGF-β, which subsequently hinders their ability to induce FOXP3 expression

**Fig 5:**
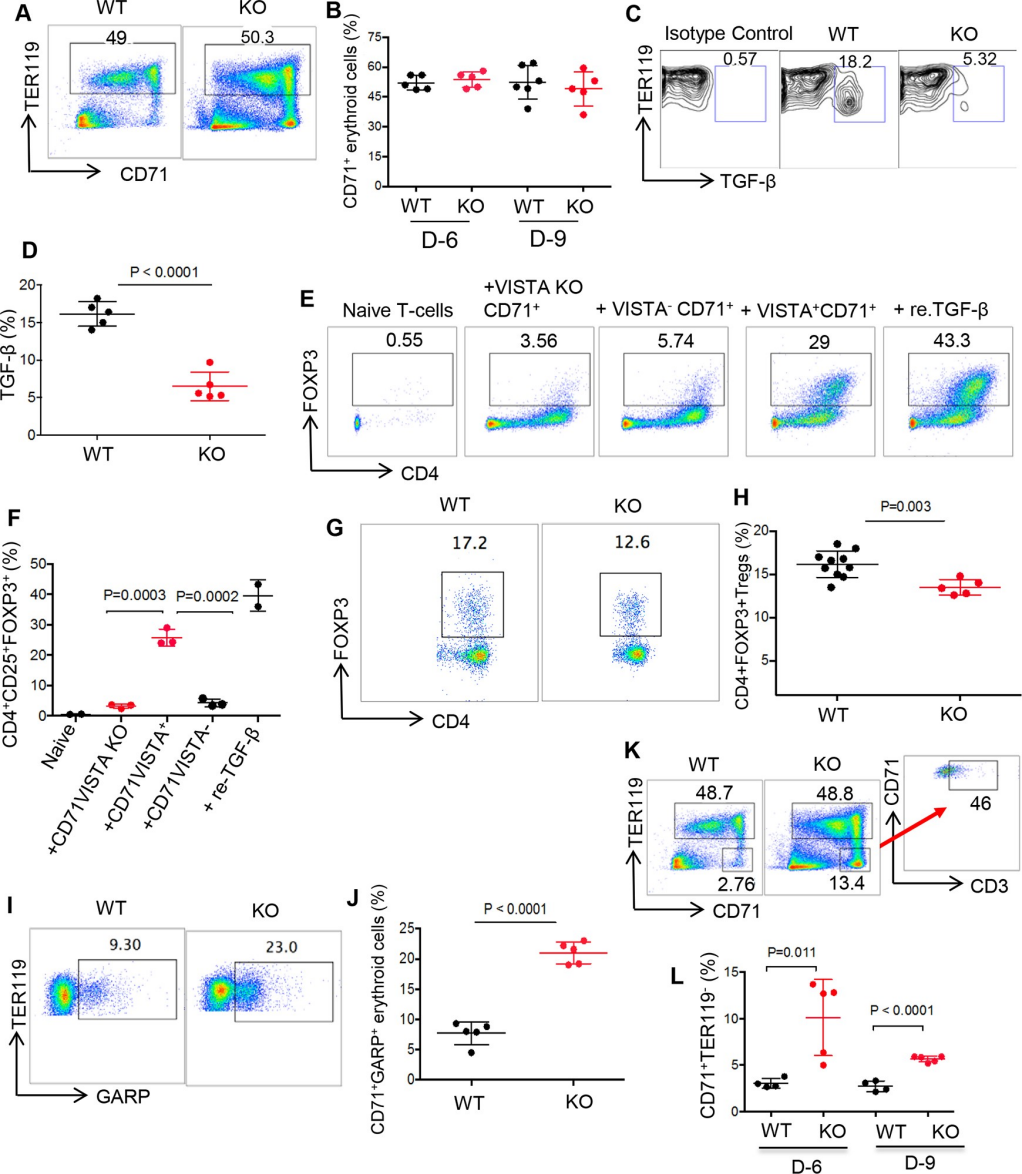
Comparison of CD71^+^ erythroid cells in VISTA KO to their counterparts in WT mice. (A). Representative plots showing frequency of CD71^+^ erythroid cells in a WT versus VISTA KO newborn mouse (day 9). (B) Cumulative data comparing the percentages of CD71^+^ erythroid cells in days 6 and 9 of WT versus KO mice. (C) Representative flow plots showing TGF-β production by a WT versus KO newborn mouse. (D) Cumulative data showing percentages of TGF-β–producing cells among CD71^+^ erythroid cells in WT and VISTA KO mice. (E) Representative flow cytometry dot plots showing percentages of iTregs in the presence of naïve T cells; CD71^+^VISTA KO, CD71^+^VISTA^−^, or CD71^+^VISTA^+^ erythroid cells; and re. TGF-β. (F) Cumulative data showing percentages of change in CD4^+^FOXP^+^ Tregs in the presence of either CD71^+^VISTA KO, CD71^+^VISTA^+^, or CD71^+^VISTA^−^ erythroid cells and re. TGF-B. (G) Representative flow plots showing percentages of Tregs in WT and VISTA KO newborn mice (day 9). (H) Cumulative data showing percentages of Tregs in WT versus VISTA KO day 9 mice. (I) Representative plots showing expression of GARP on CD71^+^ erythroid cells in WT and VISTA KO day 9 mice. (J) Cumulative data showing percentages of CD71^+^GARP^+^ cells in WT versus VISTA KO day 9 mice. (K) Representative flow plots showing CD71^+^TER119^−^ cells in WT versus VISTA KO newborns, and representative plot showing percent CD3^+^ T cells among CD71^+^TER119^−^ cells in VISTA KO mouse. (M) Cumulative data showing percentages of CD71^+^TER119^−^ cells in WT and VISTA KO mice (6 and 9 days old). Each point represents data from an individual mouse, representative of at least two independent experiments. Bar, mean ± one standard error. The underlying data can be found in S1 Data. CD71, cell-surface transferrin receptor; GARP, glycoprotein A repetitions dominant; Ig, immunoglobulin; iTreg, induced Treg; KO, knock-out; re. TGF-β, recombinant TGF-β; TER119, glycophorin A-associated protein, an erythroid-specific antigen expressed on erythrocytes; TGF-β, transforming growth factor beta; Treg, regulatory T cell; VISTA, V-domain Ig Suppressor of T Cell Activation; WT, wild type.

To better understand the function of VISTA, we investigated the role of CD71^+^ erythroid cells from VISTA KO mice for the induction of Tregs. We found no significant difference in the frequency of CD71^+^ erythroid cells between WT and VISTA KO mice at ages of 6 and 9 days (Fig 5A and 5B). However, we found impaired TGF-β production ability of CD71^+^ erythroid cells from VISTA KO compared to the WT mice (Fig 5C and 5D). Since we have shown in Fig 4C and 4D that CD71^+^VISTA^+^ erythroid cells, compared to CD71^+^VISTA^−^ erythroid cells, were superior in Treg induction, we analyzed the effects of CD71^+^ erythroid cells from VISTA KO mice compared to WT mice on FOXP3 induction in vitro. Interestingly, we found that CD71^+^ erythroid cells from VISTA KO were defective in FOXP3 induction when cocultured with naïve CD4^+^ T cells in vitro (Fig 5E and 5F). Consistently, lower Treg frequency was observed in VISTA KO compared to the WT mice (Fig 5G and 5H). Finally, the expression of GARP on CD71^+^ erythroid cells in VISTA KO and WT mice was measured. Interestingly, CD71^+^ erythroid cells from VISTA KO mice had significantly higher levels of GARP compared to the WT group (Fig 5I and 5J). Of note, an abundance of activated immune cells, CD71^+^TER119^−^, in the spleen of VISTA KO mice compared to the WT group was evident (Fig 5K and 5L). CD71 (transferrin receptor) is an activation marker and can be expressed on different immune cells including T cells [39]. It appears that these activated nonerythroid lineage cells in VISTA KO mice consist of different immune cells, but the majority were CD3^+^ T cells (Fig 5K).

### Depletion of CD71^+^ erythroid cells in vivo does not change percentages of Tregs because of the compensatory mechanism of their remaining/newly generated counterparts

**Fig 6:**
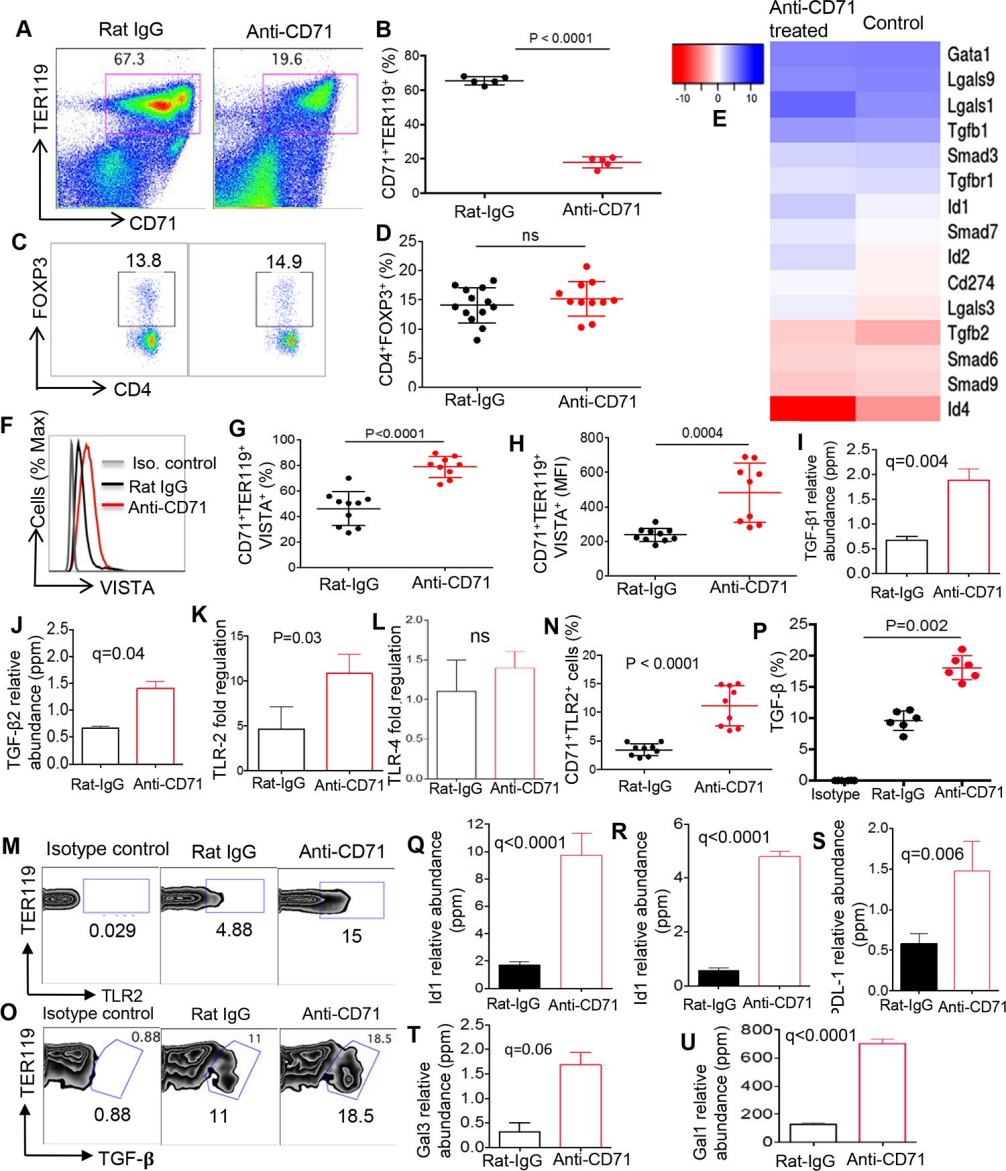
Newly generated CD71^+^ erythroid cells compensate for the loss of their counterparts. (A) Representative flow dot plots illustrating the frequency of CD71^+^ erythroid cells in the spleen of isotype (Rat-IgG) versus anti-CD71–treated newborn mice. (B) The percentage of CD71^+^ erythroid cells in the spleen of anti-CD71–treated compared to control groups in 9-day-old BALB/c mice. (C) Representative dot plots illustrating the percentages of CD4^+^FOXP3^+^ in the spleen of isotype versus anti-CD71–treated mouse. (D) Percentage of CD4^+^FOXP3^+^ in the spleen of anti-CD71–treated compared to control groups in 9-day-old BALB/c mice. (E) Gene expression of TGF-β–associated genes, galectins, and PDL-1 (CD274) in enriched CD71^+^ erythroid cells from anti-CD71–treated versus controls. (F) Representative histogram of VISTA expression on CD71^+^ erythroid cells of control versus anti-CD71–treated mouse. (G) Percentages of CD71^+^VISTA^+^ erythroid cells among splenocytes of control and anti-CD71–treated BALB/c mice. (H) The MFI of VISTA on CD71^+^ erythroid cells from the spleen of control versus anti-CD71–treated mice. (I) RNAseq data showing expression of TGF-β1 and (J) TGF-β2 mRNA in CD71^+^ erythroid cells obtained from control or anti-CD71–treated mice. (K) PCR data showing expression of TLR2, and (L) TLR4 genes in CD71^+^ erythroid cells obtained from control or treated with anti-CD71 antibody. (M) The representative zebra plots showing surface expression of TLR2 on CD71^+^ erythroid cells. (N) Cumulative data showing percentages of TLR^+^CD71^+^ erythroid cells in control versus anti-CD71–treated group. (O) Representative zebra plots showing percentage of TGF-β^+^ cells among CD71^+^ erythroid cells from controls or treated mice with anti-CD71 antibody. (P) Cumulative data showing percentages of TGF-β expressing CD71^+^ erythroid cells in rat-IgG–versus anti-CD71–treated mice. (Q) The expression of Id1, (R) Id2, (S) PDL-1, (T) Lgals3, and (U) Lgals1 mRNA levels are shown in CD71^+^ erythroid cells from either control or anti-CD71–treated mice using RNAseq. Each point represents data from an individual mouse, representative of at least three independent experiments. Bar, mean ± one standard error. The underlying data can be found in S1 Data. BALB/c, mouse strain; CD71, cell-surface transferrin receptor; FOXP3, forkhead box P3; Gal, Galectin; Gata1, erythroid transcription factor; ID, inhibitor of DNA binding; Ig, immunoglobulin; IgG, immunoglobulin G; Iso., isotype; Lgals, Galectin genes; MFI, mean fluorescence intensity; mRNA, messenger RNA; PDL-1, program death ligand-1; RNAseq, RNA sequencing; TGF-β, transforming growth factor beta; TLR, Toll-like receptor; VISTA, V-domain Ig Suppressor of T Cell Activation.

To further establish the relationship between CD71^+^ erythroid cells and Tregs, CD71^+^ erythroid cells were depleted using anti-CD71 antibody. As we have previously described, this antibody depletes 50%–60% of CD71^+^ erythroid cells in vivo [10–12]. Since CD71^+^ erythroid cells reach to the maximal levels at days 6–9 in newborns, we decided to deplete these cells by a single injection of anti-CD71 antibody (approximately 150 μg) at day 9, and 2 days later, we investigated the frequency of Tregs and CD71^+^ erythroid cells in their spleens. As shown in Fig 6A and 6B, administration of anti-CD71 antibody reduced approximately 60% of CD71^+^ erythroid cells in the spleen of mice. However, depletion of CD71^+^ erythroid cells had no significant effects on the percentage of Tregs (Fig 6C and 6D), which is in contrast to our in vitro observation (Fig 4C and 4D). Although Treg frequency was unchanged, potential changes in Treg phenotype following CD71^+^ erythroid cell depletion was studied. We observed that Tregs exhibited significantly higher MFI for CD25 and Ki67 in the absence of CD71^+^ erythroid cells, respectively (S2A and S2B Fig). In addition, significant up-regulation of PDL-1 and GARP on Tregs following anti-CD71 treatment was noted (S2C–S2F Fig). However, no changes in TIGIT or CTLA-4 expression levels in Tregs under these circumstances were observed (S2G–S2J Fig). These phenotypical changes in Tregs suggest a possible feedback when CD71^+^ erythroid cells were depleted. Since depletion of CD71^+^ erythroid cells did not impact Treg frequency in vivo, we decided to better characterize the remaining and/or newly generated CD71^+^ erythroid cells following anti-CD71 antibody administration by conducting RNAseq analysis. Interestingly, we observed that the remaining and/or newly generated CD71^+^ erythroid cells have a different transcriptional profile than their counterparts in the control animals (Fig 6E). Anti-CD71–treated animals significantly up-regulated VISTA expression compared to the IgG isotype control group in both the percentages and MFI of VISTA expression (Fig 6F–6H). In addition, CD71^+^ erythroid cell depletion led to a rise in the gene expression of both TGF-β1 and TGF-β2 by the remaining or newly produced CD71^+^ erythroid cells (Fig 6E and 6I and 6J). More importantly, we observed that CD71^+^ erythroid cells from anti-CD71-antibody–versus rat immunoglobulin G- (IgG) isotype–treated group significantly up-regulated TLR2 but not TLR4 mRNA expression levels (Fig 6K and 6L). In agreement, we found higher TLR2 surface expression on these cells compared with their native counterparts (Fig 6M and 6N). Based on these data, we decided to determine whether remaining and/or newly generated CD71^+^ erythroid cells respond to HL Lm, a TLR2 agonist, and subsequently produce more TGF-β compared to controls. Interestingly, we found this was the case, and CD71^+^ erythroid cells from the anti-CD71–treated group produced significantly higher TGF-β when stimulated with HK Lm in vitro (Fig 6O and 6P). Furthermore, we found up-regulation of inhibitors of DNA binding (ID) differentiation genes (Id1 and Id2) in CD71^+^ erythroid cells obtained from the anti-CD71–treated mice compared to the control group, which are downstream of the BMP4–VISTA pathway (Fig 6E, 6Q and 6R). Thus, higher VISTA expression may result in up-regulation of Id1 and Id2 genes, which is in line with a report indicating down-regulation of both Id1 and Id2 mRNA levels when the VISTA gene is silenced [20]. In addition, up-regulation of PDL-1, galectin-3 (Lgals3), and Lgals1 in CD71^+^ erythroid cells from the treated animals with anti-CD71 antibody versus controls was observed (Fig 6E and 6S–6U).

### Depletion of Tregs is associated with expansion of CD71^+^ erythroid cells

**Fig 7:**
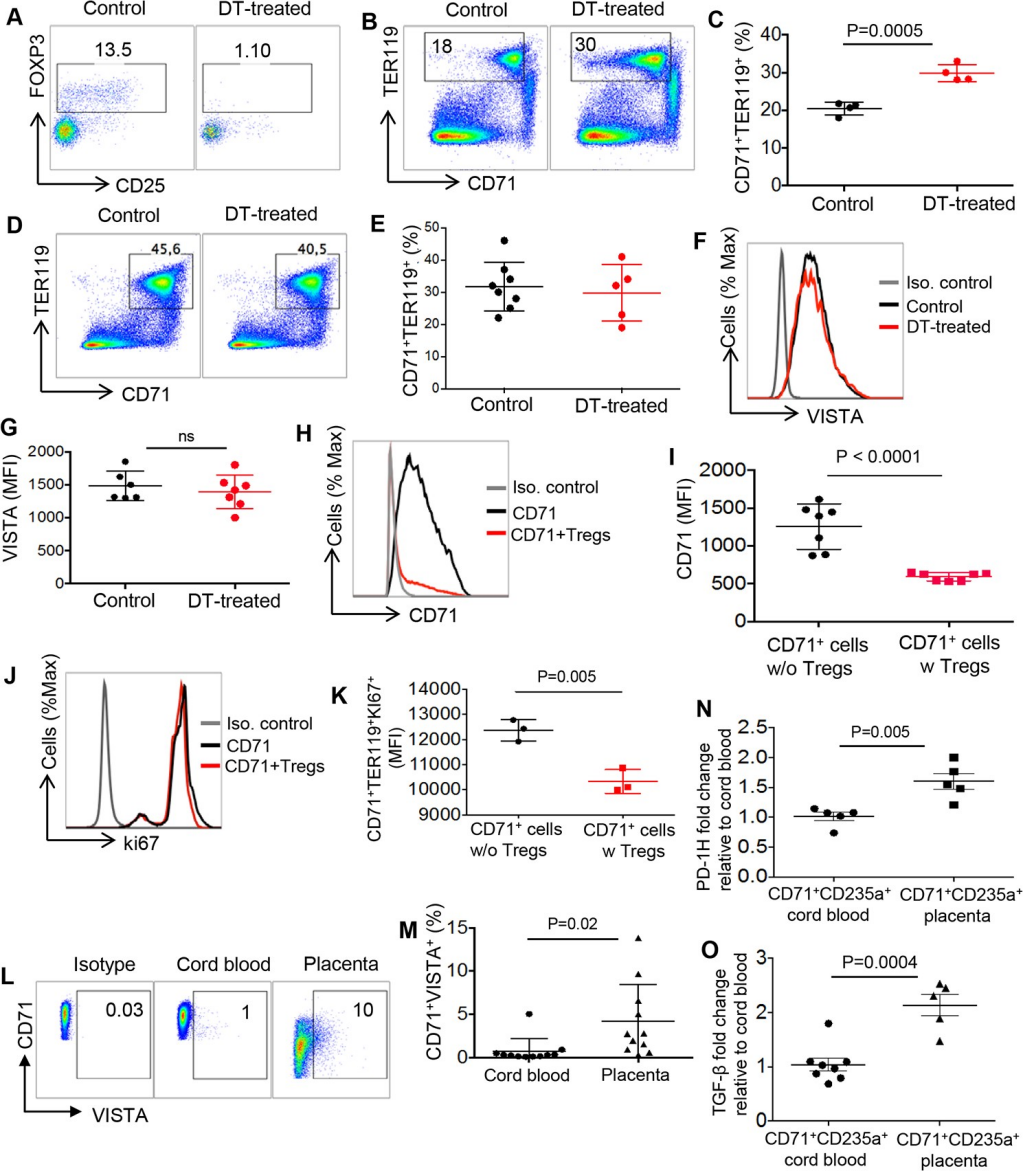
The cross-talk between Tregs and CD71^+^ erythroid cells. (A) Representative flow cytometry dot plot of Tregs in neonatal FOXP3-DTR mice either control or treated with DT. (B) Representative dot plots showing percent CD71^+^ erythroid cells in control versus DT-treated FOXP3-DTR mouse. (C) Percentages of CD71^+^ erythroid cells in control versus DT-treated mice. (D) Representative dot plots showing percent CD71^+^ erythroid cells in control versus DT-treated WT mice. (E) Percentages of CD71^+^ erythroid cells in control versus DT-treated WT mice. (F) Representative histogram plots showing expression of VISTA on CD71^+^ erythroid cells in control versus DT-treated mice. (G) Cumulative data showing MFI of VISTA on CD71^+^ erythroid cells in control versus DT-treated mice. (H) Representative histogram plots showing expression of CD71 on CD71^+^ erythroid cells in the presence and absence of Tregs. (I) Cumulative data showing MFI of CD71 on CD71^+^ erythroid cells alone or when cocultured with Tregs in vitro. (J) Representative histogram plots showing Ki67 expression on CD71^+^ erythroid cells in the presence and absence of Tregs. (K) Cumulative data showing MFI of Ki67 on CD71^+^ erythroid cells alone or upon coculture with Tregs in vitro. (L) Representative dot plots showing expression of VISTA on human cord blood and placenta CD71^+^ erythroid cells. (M) Cumulative data showing percentages of VISTA^+^ cells among human cord blood and placenta CD71^+^ erythroid cells. (N) Data showing PD-1H gene expression in enriched cord blood and placenta CD71^+^ erythroid cells. (O) Data showing TGF-β gene expression in human cord blood and placenta CD71^+^ erythroid cells. The underlying data can be found in S1 Data. CD71, cell-surface transferrin receptor; DT, diphtheria toxin; DTR, DT receptor; FOXP3, forkhead box P3; FOXP3-DTR, FOXP3 mice expressing DTR in Treg cells; Ig, immunoglobulin; Iso., isotype; Ki67, antigen KI67; MFI, mean fluorescence intensity; PD-1H, Programmed Death-1 Homologue-1; TER119, glycophorin A-associated protein, an erythroid-specific antigen expressed on erythrocytes; TGF-β, transforming growth factor beta; Treg, regulatory T cell; VISTA, V-domain Ig Suppressor of T Cell Activation; w, with; WT, wild type; w/o, without.

Despite the fact that depletion of CD71^+^ erythroid cells did not impact Treg frequency in vivo, we decided to determine what happens to the frequency of CD71^+^ erythroid cells when Tregs are depleted in newborns. Therefore, frequency of CD71^+^ erythroid cells was analyzed in the presence and absence of Tregs using FOXP3-DTR B57BL/6 mice. FOXP3-DTR mouse allows elimination of FOXP3^+^ following administration of diphtheria toxin (DT) (35 ng/g body weight) [40]. For these studies, we decided to use 11-day-old neonatal mice that had substantial percentages of Tregs. Two consecutive injections of DT at days 11 and 12, as anticipated, resulted in elimination of the majority of Tregs (Fig 7A). Subsequently, we found that elimination of FOXP3^+^ Tregs led to a significant increase in the percentages of CD71^+^ erythroid cells in these mice (Fig 7B and 7C). Finally, in order to exclude possible adverse effects of DT on the erythropoiesis in neonates, we administered DT (35 ng/g body weight) into C57BL/6 WT mice and measured the frequency of CD71^+^ erythroid cells following two consecutive treatments. As shown in Fig 7D and 7E, administration of DT did not change the frequency of CD71^+^ erythroid cells in the WT neonatal mice. To further understand the mechanisms whereby depletion of Tregs led to expansion of CD71^+^ erythroid cells, we measured VISTA expression on CD71^+^ erythroid cells. Interestingly, we did not observe any significant difference in VISTA expression levels between control and Treg-depleted groups (Fig 7F and 7G). In addition, we cocultured isolated CD71^+^ erythroid cells and Tregs to determine possible effects of Tregs on CD71^+^ erythroid cells in vitro. Interestingly, we observed that addition of Tregs to CD71^+^ erythroid cells resulted in a significant decrease in MFI of CD71 (transferrin receptor) on these erythroid cells (Fig 7H and 7I). In addition, presence of Tregs down-regulated the MFI of Ki67 on CD71^+^ erythroid cells in vitro (Fig 7J and 7K). However, no changes in the expression of other inhibitory receptors (e.g., VISTA and PDL-1) were observed (S2K–S2N Fig). These observations suggest possible cross-talk between these two cell populations during the neonatal period.

### Human CD71^+^ erythroid cells express low levels of VISTA compared to mice

We, and others, have shown that CD71^+^ erythroid cells are abundant in human cord blood and placenta tissues and coexpress CD71 and CD235α [11,41–43]. Thus, we decided to determine whether our observations were reproduceable in human samples. We found that human cord blood CD71^+^ erythroid cells express much lower levels of VISTA compared to their counterparts in mice. Interestingly, expression of VISTA was significantly higher on placenta CD71^+^ erythroid cells compared to the cord blood (Fig 7L and 7M). In addition, we performed qPCR for the expression of VISTA (PD-1H) gene in enriched cord blood and placenta CD71^+^ erythroid cells. Interestingly, we were able to detect PD-1H gene in both cord blood and placenta CD71^+^ erythroid cells. However, its expression level was significantly higher in placenta CD71^+^ erythroid cells compared to the cord blood (Fig 7N). Since human CD71^+^ erythroid cells get lysed when exposed to the perm buffer for intracellular cytokine staining, we performed qPCR on enriched CD71^+^ erythroid cells for TGF-β gene expression. We observed that both cord blood and placenta CD71^+^ erythroid cells express TGF- β; however, gene expression of this cytokine was significantly higher in placenta than cord blood CD71^+^ erythroid cells (Fig 7O).

## Discussion

In this report, we demonstrate a novel role, to our knowledge, for CD71^+^ erythroid cells in promoting the development of iTregs. Consistent with our previous studies [10,11], we have shown that CD71^+^ erythroid cells are physiologically enriched in newborns and gradually disappear by day 28. The transcriptional analysis confirms a different transcriptional profile for CD71^+^ erythroid cells in the neonatal period. This suggests that a rather drastic change in the gene expression program of CD71^+^ erythroid cells takes place early during development. However, transcriptome analysis determines that these cells consistently express some functional genes associated with inhibitory receptors and/or their ligands such as Lgals9, Lgals1, and VISTA. In agreement, we find VISTA is highly expressed on neonatal CD71^+^ erythroid cells despite some fluctuations in its expression levels throughout the first 4 weeks of life. Although Lgals9 and Lgals1 genes are highly expressed in these cells, surface expression of these molecules is substantially low on CD71^+^ erythroid cells compared to the VISTA. Here, we demonstrate that neonatal VISTA^+^CD71^+^ erythroid cells are responsible for promoting iTreg development. In contrast, VISTA^−^CD71^+^ erythroid cells and CD71^+^ erythroid cells from VISTA KO mice show significant defect in promotion of naïve CD4^+^ T cells into FOXP3^+^ iTregs when cocultured in vitro in the presence of low-dose IL-2. This observation confirms a role for VISTA in the conversion of naïve CD4^+^ T cells into iTregs. The roles of different inhibitory molecules in the development and maintenance of Tregs have been widely described. For instance, CTLA-4 is required for TGF-β–mediated induction of FOXP3^+^ Tregs from CD4^+^CD25^−^ cells [44], and Lgals9 enhances iTreg stability and function via interaction with CD44, which forms a complex with TGF-β r1 [45]. Likewise, PDL-1 promotes the induction and maintenance of iTregs [37]. Although PDL-1 signaling alone is sufficient to promote iTreg development and can promote iTreg development in the absence of TGF-β [37], this is not the case for VISTA [34]. Recently, it has been reported that compared to naïve T cells from WT mice, stimulation of naïve T cells from VISTA KO mice with anti-CD3/CD28 in the presence of TGF-β resulted in decreased induction of iTregs [34]. The induction of iTregs was nearly abolished in both WT and VISTA KO naïve T cells in the absence of TGF-β. However, the suppressive function of iTregs was not affected by the loss of VISTA [34]. Our transcriptome analysis determines that CD71^+^ erythroid cells consistently express TGF-β, which was confirmed by qPCR and flow cytometry analysis. More importantly, CD71^+^VISTA^+^ erythroid cells are the dominant TGF-β–producing cells compared to their VISTA^−^ counterparts or CD71^+^ erythroid cells from VISTA KO mice. In agreement, we find that CD71^+^VISTA^+^ erythroid cells significantly promote FOXP3 expression in naïve CD4^+^ T cells, demonstrating their TGF-β–dependent effects on the development of iTregs. This finding is further reinforced by the observation that inhibition of TGF-β abrogates FOXP3 expression in naïve CD4^+^ T cells when cocultured with CD71^+^VISTA^+^ erythroid cells. These observations demonstrate that VISTA is associated with the TGF-β overproduction phenotype in CD71^+^ erythroid cells. It is worth noting that Lgals9^+^CD71^+^ erythroid cells also contribute to TGF-β production despite their low frequency. Thus, our studies reveal a novel mechanism, to our knowledge, by which CD71^+^VISTA^+^ erythroid cells mediate immune tolerance. We have already demonstrated that CD71^+^ erythroid cells have immunosuppressive properties in the neonate [10–12] and play an essential role in fetomaternal tolerance [41,46]. CD71^+^ erythroid cells get expanded in the peripheral blood of women during the course of pregnancy; however, this was not the case when we compared the frequency of these cells in healthy women versus women with inflammatory bowel disease (IBD) [46]. Interestingly, we found reduced frequency of CD71^+^ erythroid cells in the peripheral blood of IBD women was associated with a reduction in Tregs in these patients [46]. Taken together, here we provide a novel role, to our knowledge, for CD71^+^ erythroid cells in immune hemostasis by promoting iTregs from naïve CD4^+^ T cells. However, CD71^+^ erythroid cells in human cord blood and/or placenta express much lower VISTA on their surface compared to their counterparts in neonatal mice.

Where do CD71^+^VISTA^+^ erythroid cells exert their crucial role on iTreg development? CD71^+^ erythroid cells are mostly abundant in spleen, BM, and blood but in much lower frequency in lymph nodes (approximately 5%) of newborns [10–12]. On the other hand, VISTA is widely expressed on hematopoietic and to a lesser extent on nonhematopoietic cells [19], and overexpression of VISTA is associated with a reduction in T cell activation and proliferation and with reduced cytokine production [18,19]. In addition, Wang and colleagues have shown that PD-1HIg (VISTA) promotes induction of iTregs in the presence of TGF-β [34], which is in agreement with our finding that CD71^+^VISTA^+^ erythroid cells, via TGF-β, promote development of iTregs from naïve CD4^+^ T cells. In addition, this group indicated that this effect, largely mediated via suppression of inflammatory cytokines as such VISTA signaling, prevents the conversion of iTregs to Th1 and Th17 in an inflammatory condition [34]. Although this is not investigated in our current study, we have previously reported the inhibitory effects of CD71^+^ erythroid cells on the production of Th1 and Th17 cytokines in an infection model [12]. We, and others, have shown that CD71^+^ erythroid cells from mice or human cord blood suppress cytokine production and T cell proliferation in vitro [10,11,13] and utilize arginase-2 as one of their potential immunosuppression mechanisms [10,11]. However, it should be noted that the role of CD71^+^ erythroid cells in suppression of immune responses may be more complex and possibly would work through multiple mechanisms. Although arginase-1, via dendritic cells, can promote FOXP3 induction [47], our observations do not support a role for arginase-2 in Treg development. Intriguingly, we reveal a crucial role for CD71^+^VISTA^+^ erythroid cells in the control of iTreg pool size in newborns, which may contribute to the inhibition of T cell responses. In agreement, lower Treg population and subsequently abundance of activated immune cells, especially T cells, was noted in VISTA KO mice. Although the percentages of VISTA-expressing cells among CD71^+^ erythroid cell population vary from day 3 to 28, consistently their frequency peaks at days 6–9 in different mice strains, which coincides with the similar pattern, expansion, in Treg frequency in mice. This may suggest cross-talk between CD71^+^ erythroid cells and Tregs in the newborn.

We see a dramatic reduction in the expression of VISTA on CD71^+^ erythroid cells in all three mice strains at age 12. The postnatal development of the hypothalamic-pituitary-adrenal (HPA) axis in mice occurs at two different stages. Day 12 marks the transition time when the visual stimulatory signals occur. After the stress-hyporesponsive period (day 12), mice exhibit enhanced corticosterone basal levels and a response of adrenocorticotropic hormone (ACTH) and corticosterone [48]. Some changes, such as up-regulation of VISTA, may occur following the visual stimulatory signals after day 12, when mice open their eyes. In agreement, it has been shown that corticosteroid treatment leads to the up-regulation of coinhibitory molecules such as CTLA‐4, PD‐1, CD73, and FOXP3 in a colitis model [49]. Although it is plausible that the increase in corticosteroids after day 12 enhances the expression of VISTA on CD71^+^ erythroid cells, the interaction of corticosteroids and VISTA merits further investigation.

VISTA is a crucial element of BMP4 signaling, suggesting that it acts as a BMP4 coreceptor [20]. In fact, VISTA has the highest expression levels in the BM and spleen [19], where immune cells including CD71^+^ erythroid cells are present.

In our complementary studies, we decided to find out whether depletion of CD71^+^ erythroid cells impairs Treg development in vivo. Although complete depletion of CD71^+^ erythroid cells, because of their nature (being red blood cell precursors), is impossible, their partial depletion did not impact Treg frequency in vivo. This suggests the existence of a differential mechanism in place by the remaining and/or newly generated CD71^+^ erythroid cells to compensate for the loss of their depleted siblings. In agreement, we find that the remaining and/or newly generated CD71^+^ erythroid cells express significantly higher levels of VISTA and subsequently elevated expression levels of both TGF-β1 and TGF-β2 genes. Smads 2 and 3 are receptor-regulated Smads and promote TGF-β signal via interactions with Smad 4 [50]. In contrast, Smads 6 and 7 are hindrance Smads and act to repress the TGF-β signal by competing with receptor-regulated Smads for the receptor (TβR) [51]. TGF-β overproduction can increase growth promotion by suppressing Id genes, which is the case in normal cells [50]. Conversely, in some malignant cells, unlike primary cells, Ids are not down-regulated by TGF-β [52]. Similarly, our results indicate up-regulation of Id1 and Id2 genes despite a spike in TGF-β production in CD71^+^ erythroid cells following anti-CD71 administration, suggesting that Ids can function either in concert with or opposition to TGF-β function. Although no significant difference in the Smad genes was observed in CD71^+^ erythroid cells from control versus anti-CD71–treated mice, PDL-1 was highly up-regulated in newly generated and/or remaining CD71^+^ erythroid cells post treatment. This may explain an alternative compensatory mechanism for CD71^+^PDL-1^+^ erythroid cells to promote iTreg development because PDL-1 plays a critical role in the development and maintenance of iTreg function, especially mediating immune regulation where TGF-β is present [37]. Although neonatal CD71^+^ erythroid cells do not express PDL-1, we have shown that pregnancy-induced CD71^+^ erythroid cells (either in spleen or placenta) express substantial levels of PDL-1 and/or PDL-2 [41]. Furthermore, expression of TLRs on CD71^+^ erythroid cells and changes in their expression levels following anti-CD71 treatment reveal a novel role, to our knowledge, for CD71^+^ erythroid cells in sensing pathogen-associated molecular patterns (PAMPs). The abundance of CD71^+^ erythroid cells in the newborn and their ability to recognize PAMPs illustrate a dynamic role for these cells in the neonatal period.

We also find that CD71^+^ erythroid cells attenuate the Akt signaling pathway during the conversion of naïve CD4^+^ T cells to iTregs by reducing the phosphorylation of Akt and its downstream substrate mTOR. In agreement, previous studies have shown that truncation of TCR signaling and inhibition of Akt and mTOR signaling axis are crucial for the development of iTregs [38,53,54]. This negative regulation is mediated by the neuropilin-1-semaphorin-4a axis for phosphatidylinositol 3-kinase-AKT (PI3K-Akt) [55] and of the mTOR complex 2 (mTORC2) pathway by the inositol phosphatase phosphate and tensin homolog (PTEN) [56] in Tregs.

Despite the fact that depletion of CD71^+^ erythroid cells did not influence Treg frequency in newborn mice, it potentially impacts their proliferative capacity, as shown by enhanced CD25 and Ki67 expression in Tregs in the absence of CD71^+^ erythroid cells. In addition, we decided to determine the effects of Treg depletion on the frequency of CD71^+^ erythroid cells in vivo. We find depletion of Tregs using FOXP-3-DTR mice results in a significant increase in the percentages of CD71^+^ erythroid cells. Although depletion of Tregs does not impact VISTA expression on CD71^+^ erythroid cells in vivo, we see significant reduction in the expression of CD71 and Ki67 in CD71^+^ erythroid cells when cocultured with Tregs in vitro. These observations demonstrate cross-talk between these two immunosuppressor cell populations. While revealing the molecular mechanism of this observation merits further investigations, we suggest depletion of Tregs may impact erythropoiesis. For instance, IL-2 KO mice that have lower Treg frequency experience anemia [57]. This might be due to the immune activation in the absence of Tregs and subsequently extramedullary erythropoiesis, which results in the expansion of immature red blood cells in the periphery. These data suggest that both CD71^+^ erythroid cells and Tregs, by utilizing different mechanisms, may contribute to the immune regulation in the newborn. Thus, our study highlights the important role of CD71^+^ erythroid cells in the neonatal period.

## Materials and methods

### Ethics statement

All animal experiments were carried out in accordance with the recommendations in the Guide for the Care and Use of Laboratory Animals of the Canadian Council for Animal Care. The experimental protocol was approved by the Committee on the Ethics of Animal Experiments at the University of Alberta (Protocol # AUP00001021).

### Animals

Male and female BALB/c and C57BL/6 mice were purchased from the Charles River Institute (Morrisville, NC, USA). BALB/c and C57BL/6 mice were bred together to create F1 mice. In addition, B6.129 (Cg-FOXP3 DTR) mice were purchased from The Jackson Laboratory (Bar Harbor, ME, USA). All animals were maintained and bred under pathogen-free conditions within the animal care facility at the university of Alberta. Similarly, C57BL/6 VISTA KO mice were maintained and bred under pathogen-free conditions within the animal care facility at the VA Medical Centre, White River Junction, VT, USA.

### CD71^+^ erythroid cells and Treg depletion

For in vivo depletion, purified anti-CD71 (8D3) and rat IgG2a isotype control antibodies were administered i.p. (150–200 µg) at day 6, and 2 days later, spleens were harvested for immunological assays.

For Treg depletion, FOXP3-DTR neonatal mice (9 days old) were injected i.p. with DT (Sigma-Aldrich), 35 ng/g body weight, before sample collection at day 11.

### Flow cytometry analysis

Fluorophore or biotin-conjugated antibodies with specificity to mouse cell antigens and cytokines were purchased from eBioscience (Waltham, MA, USA) or BD Biosciences (San Jose, CA, USA). Specifically, the following antibodies were used for mouse studies: anti-CD71 (C2), anti-VISTA (MIH64, MIH63), anti-LAP (TW7-16B4), anti-CD3 (500A2), anti-CD4 (RM4-5), anti-CD62L (MEL-14), anti-CD44 (5035–41), anti-TER-119 (TER-119), anti-CD73 (Ty/23), anti-CD39 (24DMS1), anti-PDL-1 (MIH5), anti-TIGIT (1G9), anti-Helios (22F6), anti-CD25 (PC61.5), anti-FOXP3 (FJK-165), GARP (YGIC86), IgG1 K ISO control (P3.6.2.8.1), anti-CD281(TLR1, eBioTR23), and anti-CD284 (TLR4, MT5510). Recombinant mouse TGF-β1, anti-Lgals9 (RG-395), and anti-CD282 (TLR2, T2.5) were purchased from Biolegend (San Diego, CA, USA), and TGF-β1 blocker was purchased from Sigma-Aldrich (St. Louis, MO, USA). Anti-Lgals1 (EPR3205) and the secondary antibody (A31572) were purchased from Abcam (Cambridge, UK). Human antibodies against CD71 (MA712) and CD235a (HIR2) from BD and VISTA (B7H5DS8) were purchased from eBioscience. Live dead staining kits were purchased from Invitrogen (Carlsbad, CA, USA). Purified NA/LE Hamster anti-mouse CD3e(145-2C11), CD28(37.51), phospho-Akt, and phospho-mTOR were purchased from BD Bioscience. Surface or intracellular staining were performed as we have described elsewhere [11,16]. TGF-β levels were measured following intracellular staining using Latency Associated Protein (LAP). Paraformaldehyde fixed cells were acquired using a BD LSR Fortessa-SORP or Fortessa-X20 (BD Bioscience). Data analysis was performed by using FlowJo software (version 10).

### Cell isolation and cell culture

Mice spleens were processed, and in order to obtain single-cell suspensions, spleen samples were ground between sterile frosted glass slides in 7 ml of 1× RBC lysis buffer and then filtered through nylon mesh. Cells were resuspended and processed in Dulbecco’s Modified Eagle’s Medium (DMEM)—high glucose (Sigma) with 1% penicillin/streptomycin (Sigma), 10% FBS (Sigma), and nonessential amino acids (Sigma). For cell enrichment, CD71^+^ erythroid cells were enriched as we previously described elsewhere [10,11] with a purity typically exceeding 95% (S2O Fig). Furthermore, isolated CD71^+^ erythroid cells were labeled with PE-conjugated anti-VISTA mAb (MIH63), followed by anti-PE microbeads (Miltenyi Biotec), and passed through MACS separation columns (Miltenyi Biotec). VISTA^+^ and VISTA^−^ were isolated based on positive and negative selections, respectively. The purity of VISTA^+^ and VISTA^−^ cells was approximately 95% as shown in S2P Fig. Naive CD4^+^ T cells were isolated from the adult mice spleens according to the manufacturing instruction (Stemcell Technologies) with purity > 90% (S2Q Fig).

### Assessment of Treg induction and their characterization

Naïve CD4^+^ T cells were cultured in 96-well plates in the presence of either CD71^+^/VISTA^+^ or CD71^+^/VISTA^−^ erythroid cells from WT mice and CD71^+^VISTA^−^ from VISTA KO mice, supplemented with recombinant IL-2 (100 IU/ml), and stimulated with soluble anti-CD3 (3 μg/ml) and anti-CD28 (1 μg/ml) antibodies. Mouse recombinant TGF-β1 (Biolegend) and TGF-β1 blocker (Sigma) were also used as positive and negative controls, respectively. Cultures were analyzed 4–5 days later for FOXP3 induction.

For functional assays, Tregs were isolated from either splenocytes of adult mice or following generation (coculture of naïve CD4^+^ T cells with neonatal CD71^+^VISTA^+^ erythroid cells) using Treg isolation kit (Stemcell Technologies, Vancouver, Canada), then cocultured with effector T cells at different ratios for proliferation assay using CFSE dye (Thermo Fisher Scientific, Waltham, MA, USA). Naïve CD4^+^ T cells were cultured for 24 h in 96-well plates with or without CD71^+^ erythroid cells in the presence of recombinant IL-2 (100 IU/ml) and anti-CD3 (3 μg/ml) and anti-CD28 (1 μg /ml). Then, phospho-AKT and phospho-mTOR were performed according to the manufacturing instruction.

### Gene expression assay

Enriched CD71^+^ erythroid cells were subjected to total RNA extraction in TRIZOL reagent (Invitrogen) using the RNeasy kit (Qiagen, Venlo, The Netherlands). A Nano-Drop ND-1000 Spectrophotometer (NanoDrop Technologies, Wilmington, DE, USA) was used to check the quantity and quality of RNA for each sample. Usually, 500 ng of the isolated RNA was reverse transcribed employing a QuantiTect Reverse Transcription kit (Qiagen). Gene expression of PD-1H (VISTA), TGF-β, and TLRs was calculated by the 2^−ΔΔ*Ct*^ method. Glyceraldehyde phosphatidyl hydrogenase (GAPDH) was used as a housekeeping gene for normalization of the cDNA levels. Samples collected at day 1 postnatal were used as the calibrator samples. The negative controls contained water or reverse-transcription negative RNA instead of template DNA.

### RNAseq

RNAseq libraries were constructed from 500 ng of total RNA using the TruSeq RNA Library Prep kit v2 (Illumina, San Diego, CA, USA) according to the manufacturer’s instructions at The Applied Genomic Core (TAGC), University of Alberta. Libraries were sequenced on a NextSeq 500 instrument (Illumina) using a 75-bp paired-end protocol at an approximate depth of 12 M paired-end reads per sample. Transcripts abundance was quantified using Kallis [58] and 100 bootstraps. Differential expression analysis was conducted using Sleuth [59]. Data postprocessing was carried out with in-house R scripts.

### Statistical analysis

Statistical comparison between various groups was performed by the *t* test using PRISM software. In the gene expression assay, differences between adult group and newborns at different time points were evaluated using one-way ANOVA, followed by Tukey’s test for multiple comparisons. Results are expressed as mean ± SEM. *p* value < 0.05 was considered as statistically significant.

## Supporting information

S1 Fig (A) Representative flow cytometry dot plots showing CD71^+^ erythroid cells in the spleen of BALB/c mice. (B) The percentages of CD71^+^ erythroid cells in spleen of BALB/c mice, (C) C57BL/6, and (D) F1 mice at different ages, respectively. (E) Percentages of VISTA^+^CD71^+^ erythroid cells in spleen versus BM. (F) Representative dot plots showing percent CD71^+^ erythroid cells in a newborn and an adult mouse. (G) Percentages of VISTA-expressing CD71^+^ erythroid cells in the spleens of female versus male mice. (H) Representative plots showing coexpression of Lgals9 and VISTA and (I) and Lgals1 and VISTA on CD71^+^ erythroid cells. (J) Representative plots showing coexpression of VISTA and GARP on CD71^+^ erythroid cells. (K) Cumulative data showing percentages of GARP^+^ cells among CD71^+^ erythroid cells. (L) Absolute number of Tregs at different ages of BALB/c mice are shown. (M) Cumulative data showing induction of Tregs in the presence of total CD71^+^ erythroid cells and different concentrations of L-arginine in vitro. The underlying data can be found in S2 Data. BM, bone marrow; CD71, cell-surface transferrin receptor; F1, filial 1 hybrid mice; GARP, glycoprotein A repetitions predominant; Ig, immunoglobulin; Lgals1, galectin-1; Lgals9, galectin-9; Treg, regulatory T cell; VISTA, V-domain Ig Suppressor of T Cell Activation.
(TIF)

S2 Fig (A) Cumulative data showing MFI of CD25 and (B) MFI of Ki67 among Tregs from control (rat IgG) and anti-CD71–treated newborn mice. (C) Representative histogram plots showing expression of PDL-1 on Tregs and (D) cumulative data showing MFI of PDL-1 on Tregs from control versus anti-CD71–treated mice. (E) Representative histogram plots showing expression of GARP on Tregs and (F) cumulative data showing MFI of GARP on Tregs in control versus anti-CD71–treated mice. (G) Representative histogram plots showing expression of TIGIT and (H) cumulative MFI of TIGIT on Tregs in control versus anti-CD71–treated mice. (I) Representative histogram plots showing expression of CTLA-4 and (J) cumulative MFI of CTLA-4 on Tregs in control versus anti-CD71–treated mice. (K) Representative histogram plots showing expression of VISTA and (L) cumulative data on MFI of VISTA on CD71^+^ erythroid cells alone or once cocultured with Tregs in vitro. (M) Representative histogram plots showing expression of PDL-1 on CD71^+^ erythroid cells and (N) cumulative data on MFI of PDL-1 on CD71^+^ erythroid cells in the presence or absence of Tregs in vitro. (O) Representative dot plot showing purity of CD71^+^ erythroid cells pre- and postenrichment. (P) Representative histogram plots showing purity of CD71^+^VISTA− and CD71^+^VISTA^+^ erythroid cells postenrichment. (Q) Representative dot plot indicating purity of naïve CD4^+^ T cells pre- and postenrichment. Each point represents data from an individual mouse, representative of at least two independent experiments. Bar, mean ± one standard error. The underlying data can be found in S2 Data. CD71, cell-surface transferrin receptor; CTLA-4, cytotoxic T-lymphocyte-associated protein 4; GARP, glycoprotein A repetitions predominant; Ig, immunoglobulin; IgG, immunoglobulin G; Ki67, antigen KI67; MFI, mean fluorescence intensity; PDL-1, program death ligand-1; TIGIT, T cell immunoreceptor with Ig and ITIM domains; Treg, regulatory T cell; VISTA, V-domain Ig Suppressor of T Cell Activation.
(TIF)

S1 Data (XLSX)

S2 Data (XLSX)

## Abbreviations

ACTH: adrenocorticotropic hormone
Akt: protein kinase B
Ankrd44: ankyrin repeat domain 44
APC: antigen-presenting cell
BM: bone marrow
BMP4: Bone Morphogenic Protein-4
c10orf54: another name for VISTA
CD71: cell-surface transferrin receptor
CFSE: carboxyfluorescein succinimidyl ester
Con-A: Concanavalin A
CTLA-4: cytotoxic T-lymphocyte–associated protein 4
DD1α: another name for VISTA gene
Dies1: another name for VISTA gene
DMEM: Dulbecco’s Modified Eagle’s Medium
DT: diphtheria toxin; DTR, diphtheria toxin receptor
F1: filial 1 hybrid mice
FOXP3: forkhead box P3
Foxp3-DTR: mice expressing DTR in Treg cells
GAPDH: glyceraldehyde phosphatidyl hydrogenase
GARP: glycoprotein A repetitions predominant; Gata1, erythroid transcription factor
Gi24: another name for VISTA
GVHD: Graft Versus Host Disease
HK: heat killed; HPA, hypothalamic-pituitary-adrenal
IBD: inflammatory bowel disease
ID: inhibitors of DNA binding
Ig: immunoglobulin
IgG: immunoglobulin G
IL-23: Interleukin-23
Iso.: isotype
ITIM: immunoreceptor tyrosine-based inhibition motif
iTreg: induced Treg
ITSM: immunoreceptor tyrosine-based switch motif
Ki67: antigen KI67
KO: knock-out; LAG-3, lymphocyte activation 3
Lgals: galectin genes
LAP: Latency Associated Protein
Lm: *Listeria monocytogenes*
MFI: mean fluorescence intensity
miR-125a: microRNA-125a
mRNA: messenger RNA
mTOR: The mechanistic target of rapamycin
mTORC2: the mechanistic target of rapamycin complex 2
NK: natural killer
Pam_3_CSK_4_: N-palmitoyl-S-dipalmitoylglyceryl Cys-Ser-(Lys) 4
PAMP: pathogen-associated molecular pattern
PC: principal component
PCA: principal component analysis
PD-1: program death-1
PD-1H: Programmed Death-1 Homologue-1
Pdcd1lg2: Programmed Cell Death 1 Ligand 2 gene
PDL-1: program death ligand-1
phospho-Akt: phosphorylated-Akt
phospho-mTOR: phosphorylated-mTOR
PI3K-Akt: phosphatidylinositol 3-kinase-AKT
Ppbp: pro-platelet basic protein gene
PTEN: phosphatase and tensin homolog
qPCR: quantitative polymerase chain reaction
r1: receptor 1
re. TGF-β: recombinant TGF-β
RNAseq: RNA sequencing
RT-PCR: reverse transcription polymerase chain reaction
SISP1: another name for VISTA
TAGC: The Applied Genomic Core
Teff: effector T cell
TER119: glycophorin A-associated protein
TGF-β: transforming growth factor beta
Th1: T helper-1
TIGIT: T cell immunoreceptor with Ig and ITIM domains
TIM-3: T-cell immunoglobulin and mucin-domain containing-3
TLR: Toll-like receptor
Treg: regulatory T cell
TβR: TGF-β receptor
VISTA: V-domain Ig Suppressor of T Cell Activation
Vsir: another name for VISTA
WT: wild type
