# Supplementary figures and images for: CD71+VISTA+ erythroid cells promote the development and function of regulatory T cells through TGF-β
Source: PLoS Biol. 2018 Dec 14;16(12):e2006649. doi: 10.1371/journal.pbio.2006649 (PMC6310287; doi:10.1371/journal.pbio.2006649)

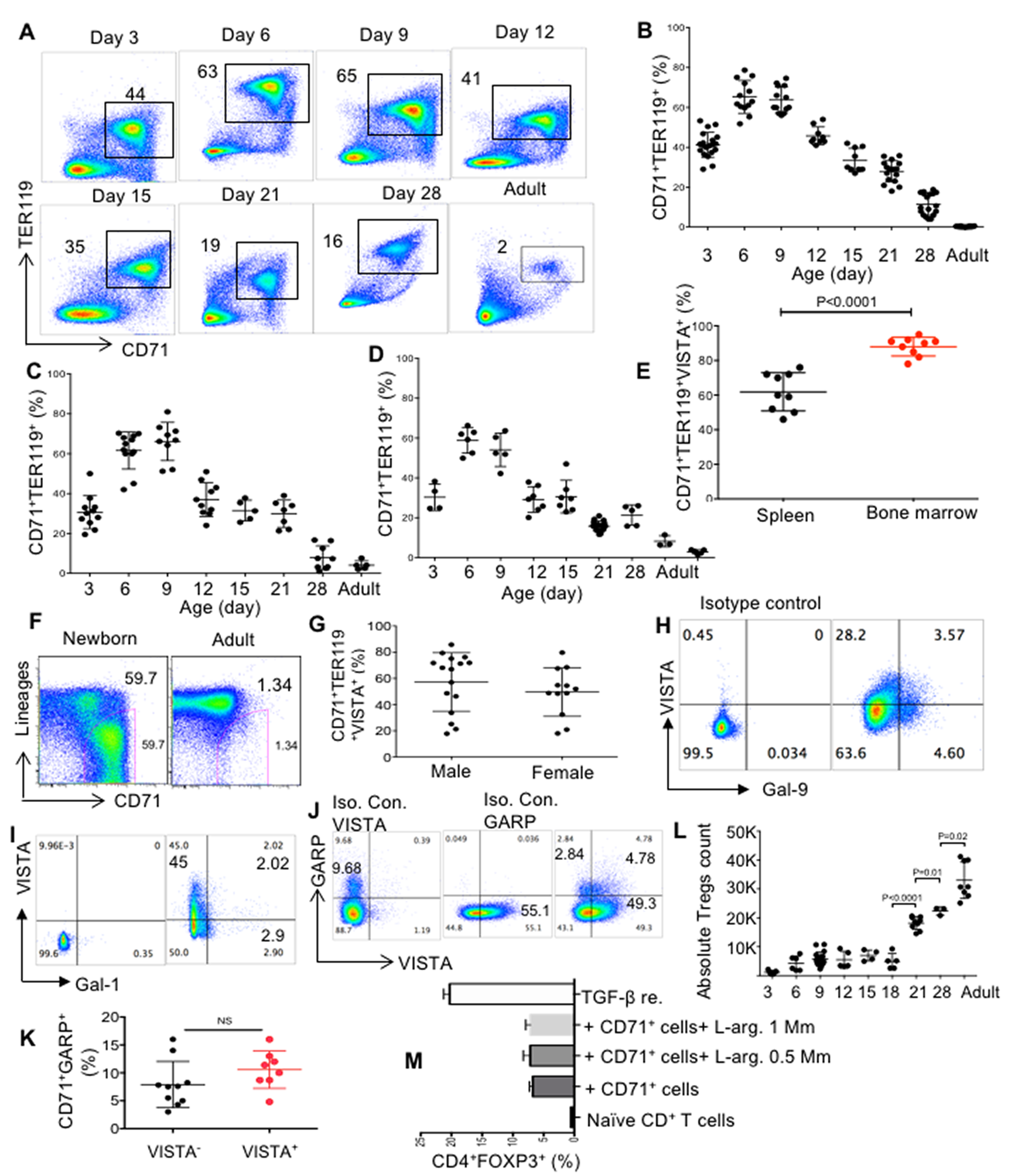

Supplement: S1 Fig — (A) Representative flow cytometry dot plots showing CD71+ erythroid cells in the spleen of BALB/c mice. (B) The percentages of CD71+ erythroid cells in spleen of BALB/c mice, (C) C57BL/6, and (D) F1 mice at different ages, respectively. (E) Percentages of VISTA+CD71+ erythroid cells in spleen versus BM. (F) Representative dot plots showing percent CD71+ erythroid cells in a newborn and an adult mouse. (G) Percentages of VISTA-expressing CD71+ erythroid cells in the spleens of female versus male mice. (H) Representative plots showing coexpression of Lgals9 and VISTA and (I) and Lgals1 and VISTA on CD71+ erythroid cells. (J) Representative plots showing coexpression of VISTA and GARP on CD71+ erythroid cells. (K) Cumulative data showing percentages of GARP+ cells among CD71+ erythroid cells. (L) Absolute number of Tregs at different ages of BALB/c mice are shown. (M) Cumulative data showing induction of Tregs in the presence of total CD71+ erythroid cells and different concentrations of L-arginine in vitro. The underlying data can be found in S2 Data. BM, bone marrow; CD71, cell-surface transferrin receptor; F1, filial 1 hybrid mice; GARP, glycoprotein A repetitions predominant; Ig, immunoglobulin; Lgals1, galectin-1; Lgals9, galectin-9; Treg, regulatory T cell; VISTA, V-domain Ig Suppressor of T Cell Activation. (TIF) [file pbio.2006649.s001.tif]

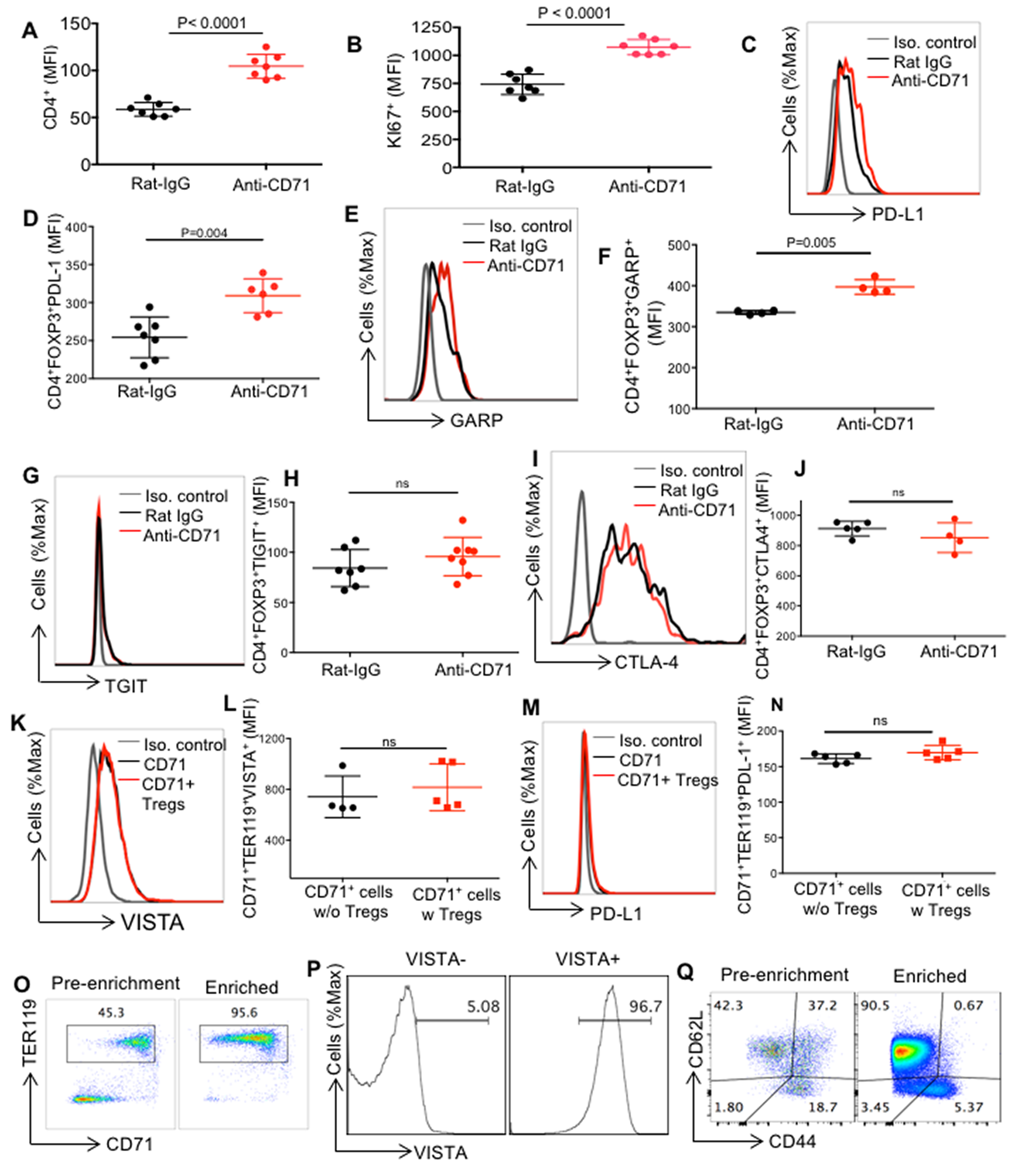

Supplement: S2 Fig — (A) Cumulative data showing MFI of CD25 and (B) MFI of Ki67 among Tregs from control (rat IgG) and anti-CD71–treated newborn mice. (C) Representative histogram plots showing expression of PDL-1 on Tregs and (D) cumulative data showing MFI of PDL-1 on Tregs from control versus anti-CD71–treated mice. (E) Representative histogram plots showing expression of GARP on Tregs and (F) cumulative data showing MFI of GARP on Tregs in control versus anti-CD71–treated mice. (G) Representative histogram plots showing expression of TIGIT and (H) cumulative MFI of TIGIT on Tregs in control versus anti-CD71–treated mice. (I) Representative histogram plots showing expression of CTLA-4 and (J) cumulative MFI of CTLA-4 on Tregs in control versus anti-CD71–treated mice. (K) Representative histogram plots showing expression of VISTA and (L) cumulative data on MFI of VISTA on CD71+ erythroid cells alone or once cocultured with Tregs in vitro. (M) Representative histogram plots showing expression of PDL-1 on CD71+ erythroid cells and (N) cumulative data on MFI of PDL-1 on CD71+ erythroid cells in the presence or absence of Tregs in vitro. (O) Representative dot plot showing purity of CD71+ erythroid cells pre- and postenrichment. (P) Representative histogram plots showing purity of CD71+VISTA− and CD71+VISTA+ erythroid cells postenrichment. (Q) Representative dot plot indicating purity of naïve CD4+ T cells pre- and postenrichment. Each point represents data from an individual mouse, representative of at least two independent experiments. Bar, mean ± one standard error. The underlying data can be found in S2 Data. CD71, cell-surface transferrin receptor; CTLA-4, cytotoxic T-lymphocyte-associated protein 4; GARP, glycoprotein A repetitions predominant; Ig, immunoglobulin; IgG, immunoglobulin G; Ki67, antigen KI67; MFI, mean fluorescence intensity; PDL-1, program death ligand-1; TIGIT, T cell immunoreceptor with Ig and ITIM domains; Treg, regulatory T cell; VISTA, V-domain Ig Suppressor of [file pbio.2006649.s002.tif]
